# Shaping electrospray plume with convergent sound beams

**DOI:** 10.1016/j.isci.2025.112388

**Published:** 2025-04-08

**Authors:** Yu-Hao Chen, Min-Min Hung, Pawel L. Urban

**Affiliations:** 1 Department of Chemistry, National Tsing Hua University, Hsinchu 300044, Taiwan

**Keywords:** Natural sciences, Acoustics, Applied sciences

## Abstract

Electrospray ionization (ESI) is one of the mainstream ionization techniques for mass spectrometry (MS) nowadays. Here, we present a new variant of ESI-MS setup, in which an electrospray emitter points to the mass spectrometer’s orifice while the collimated sound (from one or four woofers) propagates orthogonally to the plume and MS inlet axis. First, we investigated deflection of electrospray microdroplets of different sizes by the sound waves using high-speed imaging. Low-frequency sound influences microdroplets of different sizes to a varied extent enabling partial separation. Second, we conducted a series of on-line experiments using a range of peptides, polymers, and amino acids. The influence of sound on microdroplet motion is more pronounced at higher amplitudes and lower frequencies of the signal used to generate the sound stream. Our findings provide a way to modulate electrospray process and can impact the application areas of electrospray including MS and materials science.

## Highlights

• Electrospray ionization is widely used to produce ions for mass spectrometry
• Electrospray droplets can be manipulated by convergent sound beams
• Sound deflects electrospray droplets, as revealed by high-speed imaging
• Ion currents of some organic compounds can be increased this way

## Graphical abstract

**Figure:**
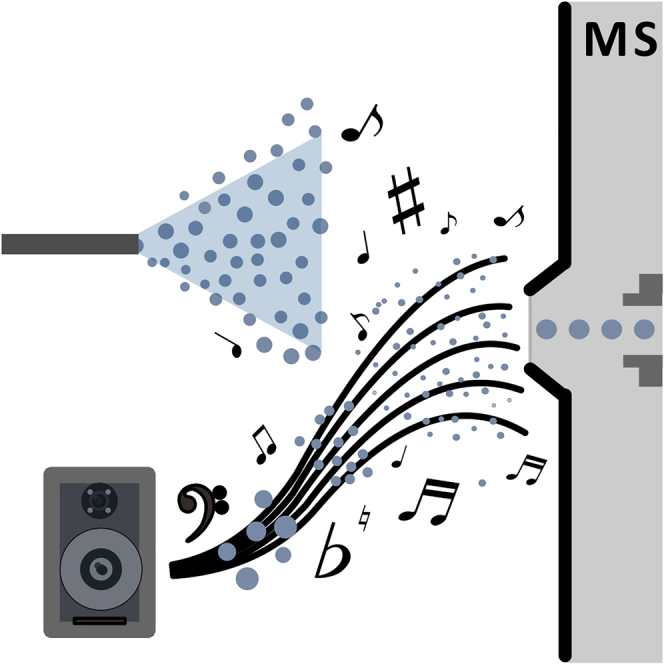

## Introduction

Electrospray ionization (ESI) has proven to be a powerful ion source for a wide range of mass spectrometry (MS) applications.^1^^,^^2^^,^^3^^,^^4^ It is a soft ionization technique, suitable for analysis of polar and non-volatile analytes over an extensive mass range. One of the most attractive features of ESI is its high compatibility with liquid chromatography (LC), which makes analysis of complex samples by LC-MS possible.^5^^,^^6^^,^^7^ The sensitivity of ESI-MS is influenced by ionization efficiency and transmission efficiency.^8^ Ionization efficiency of ESI is related to the thermodynamic properties of analytes, including nonpolar surface area, free energy of solvation, and gas-phase proton affinities.^9^ The ionization efficiencies of various compounds span over a million-fold range.^10^ Because only a minor fraction of analytes can successfully pass through a narrow MS inlet, which is required to maintain vacuum,^11^ ESI-MS systems also suffer from low ion transmission efficiencies. Therefore, transmission efficiencies of conventional ESI range from ∼0.00001% to 0.1%.^8^^,^^12^^,^^13^ In order to address this problem, certain improvements of ESI setup have been proposed.

There exist variants of ESI that show superior performance. For instance, in nanoelectrospray ionization (nanoESI), the inner diameter of the emitter can be in the order of 1–2 μm, and the flow rate can be as low as ∼ 20 nL min^−1^.^14^ These characteristics result in the generation of very small initial droplets.^14^ In addition to different variants of ESI, the incorporation of focusing components can help to transmit the contents of electrospray plume. An ion funnel—composed of a series of cylindrical ring electrodes with different inner diameters—is used to concentrate the spatially dispersed ion cloud.^15^ Ring electrodes are also employed to focus the electrospray plume.^16^^,^^17^ By adjusting the voltage applied to the ring electrode, it is possible to influence the electrospray plume.^18^ Kottke et al. introduced an ESI-MS interface that utilizes a swirling flow to enable the selection of smaller droplets from the ESI plume toward the MS inlet via the inertial separation.^19^ Wang et al. demonstrated the effect of low-frequency sound wave (50–350 Hz) on the ESI process.^20^ In the so-called low-frequency-sound-modulated ESI, a woofer was directed toward the MS inlet, while ESI capillary was positioned vertically and orthogonally to both sound path and ion-transfer line.^20^ This setup enabled redirection of electrospray microdroplets toward the orifice of the mass spectrometer, influencing analyte signal intensities.^20^ We believe the ability to modulate MS signals was restricted by the chosen (orthogonal) geometry of the electrospray setup.

In the present study, the ESI capillary is coaxial to the MS inlet while the woofer is positioned perpendicularly to the axes of both ESI capillary and MS inlet, which makes a substantial qualitative and quantitative difference relative to the previous setups. We aimed to investigate the effect of a sound beam on trajectories of microdroplets of different sizes and MS signals. We also aimed to collimate electrospray plume using multiple beams of sound relayed on the plume circumference to alter sensitivity and selectivity of ESI-MS analysis.

## Results and discussion

**Figure 1:**
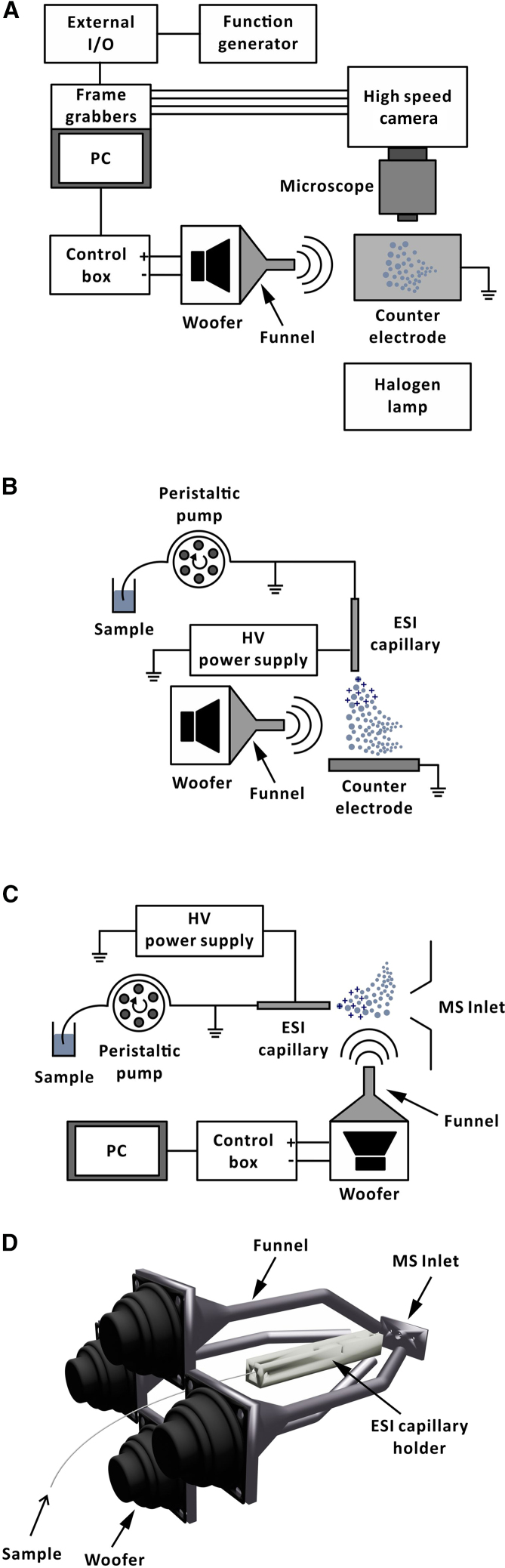
Schematic diagram of experimental setups for on-axis electrospray ionization with orthogonal sound (A) Top view of the offline setup with one large woofer; (B) side view of the offline setup with one large woofer; (C) online setup with one large woofer; (D) visualization of the setup with four small woofers.

This study relies on custom-developed experimental setups, which are detailed in the STAR Methods in the following, including the key resources table, and illustrated in Figures 1 and S1–S4.

### High-speed imaging of charged microdroplets deflected by sound

**Figure 2:**
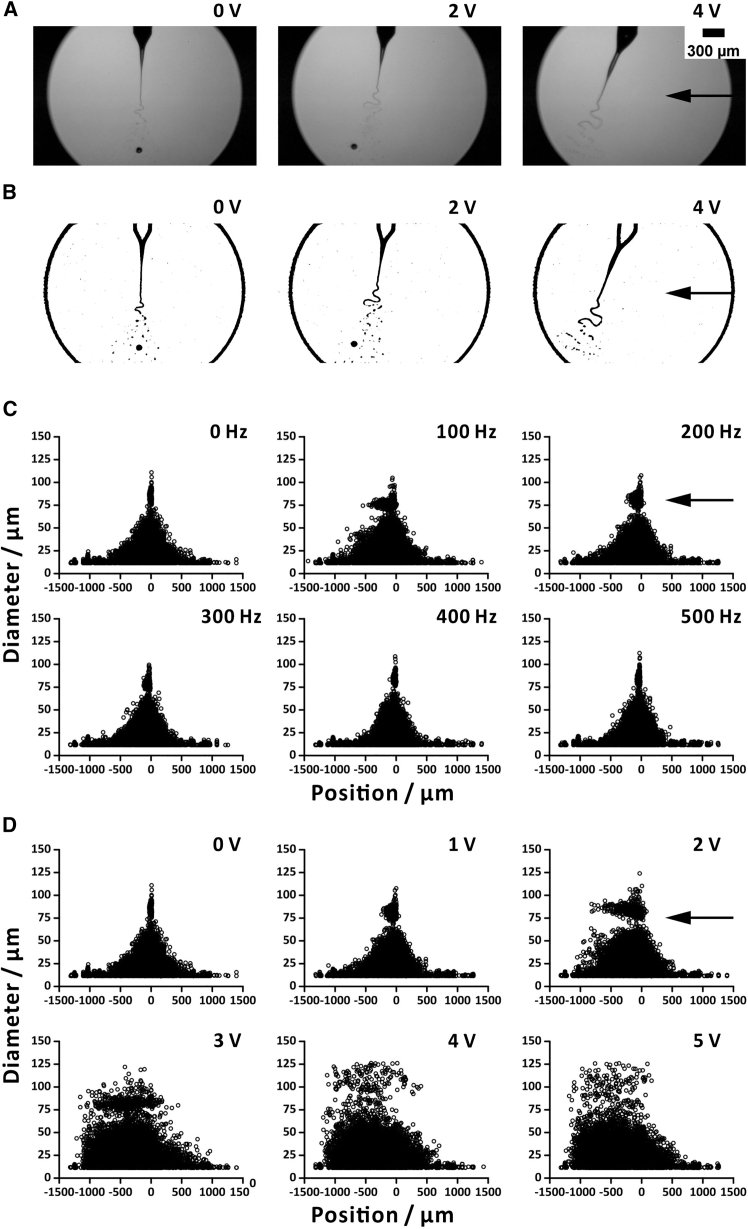
High-speed images of electrospray plume with different amplitudes of low-frequency (200 Hz) sound (A) Raw images and (B) processed images. Correlations between diameters of microdroplets and the distance between the ESI capillary axis and microdroplets. Effect of (C) frequency and (D) amplitude of sound. Voltage applied to the ESI capillary was 3.8 kV. Flow rate was 50 μL min^−1^. Default frequency, 200 Hz; default amplitude, 1 V. Each plot is based on processing 900 images. Black arrows indicate the direction of sound. Scale bar: 300 μm. See also Video S1.

To investigate the influence of sound waves on microdroplets, we utilized high-speed imaging, which allowed us to gain an insight into the positions of microdroplets within electrospray plume exposed to sound (Figures 2A and 2B, Video S1). Microdroplets of ∼75 μm were only deflected by less than 500 μm, whereas microdroplets of ∼50 μm were deflected by up to ∼800 μm under a low-frequency sound field (100 Hz, 1 V; Figure 2C). However, when the sound frequency with the same amplitude was greater than 200 Hz, the deflection of microdroplets was almost unobservable. When the signal amplitudes were varied, we found that increasing the sound amplitude would lead to a more apparent deflection of microdroplets (Figure 2D). It can be seen in the images obtained in the presence of a strong sound (200 Hz, 5 V), the microdroplets of ∼50–75 μm can be deflected by up to ∼1,000 μm (Figure 2D). Based on the previous theoretical predictions, the movement of droplets in a sound field manifests itself as an oscillatory state, which is influenced mainly by the frequency and sound pressure level (SPL) of the sound waves, as well as the size of the droplets.^21^^,^^22^^,^^23^ However, during the ESI process, there are kink instabilities,^24^ which complicate attributing droplet motion solely to the sound field.

Video S1. Influence of sound (at different amplitudes) on electrospray droplets, related to Figure 2

**Figure 3:**
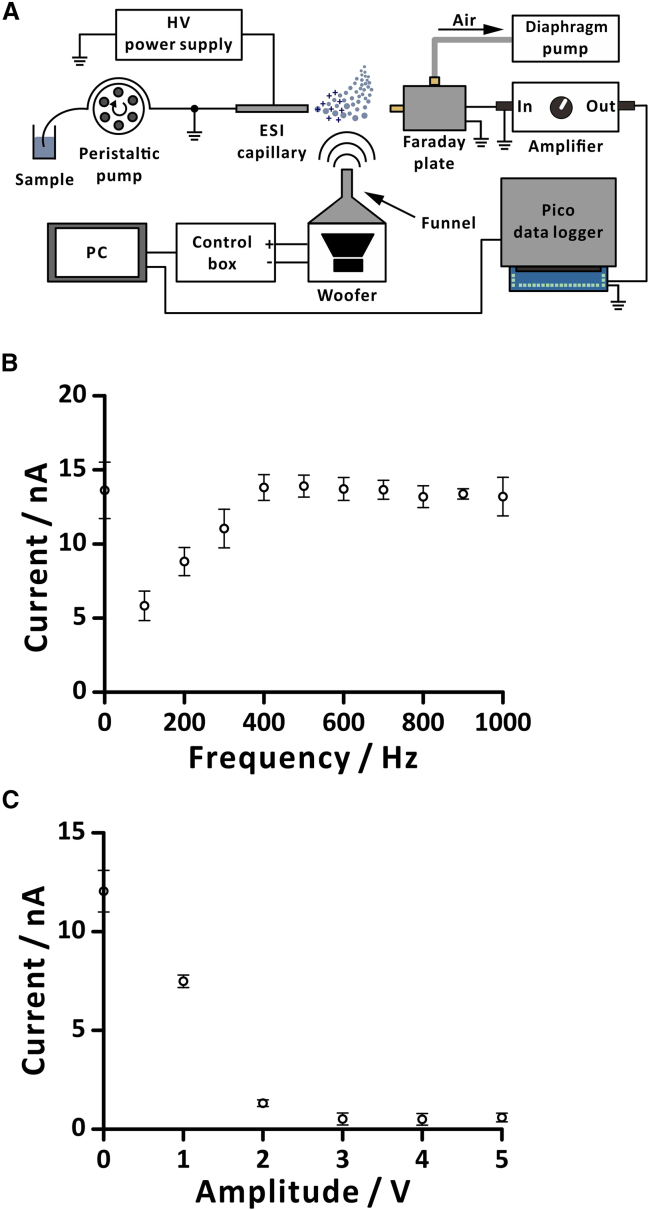
Offline measurement of ion current with orthogonal sounds (A) Schematic of the setup; (B) ion current vs. frequency with sinusoidal voltage amplitude (1 V); (C) ion current vs. amplitude with sinusoidal voltage frequency (200 Hz). Sample: 25% (v/v) methanol in water. Voltage applied to the ESI capillary was 3.8 kV. The flow rate was 50 μL min^−1^. Magnification factor of the amplifier (V/A): 10^7^. Sampling interval of the data logger was 60 ms. The ion current was averaged from 0 to 30 s. Replicates, *n* = 3. Data are represented as mean ± SD.

In a separate test, we utilized a Faraday plate detector to measure ion current of electrospray plume with side exposure to sound beam (Figure 3A). We observed that the low-frequency sound (100–300 Hz) yielded lower ion currents than high-frequency sound or the condition without sound (Figure 3B). For the measurement of ion currents with different amplitudes, the ion currents dramatically dropped when high amplitude signals were used (2–5 V; Figure 3C). The higher the amplitudes of the sound, the lower the ion currents were measured. It should be noted that higher sound amplitudes exhibited a greater deflection of microdroplets than that observed for lower sound amplitudes, resulting in lower ion currents. However, the diameters of most droplets were less than ∼50 μm (Figure S5). Moreover, when sound amplitudes were higher than 3 V, there was no significant change in ion currents. This is because the SPL reaches its maximum when the amplitudes of the signal supplied to the input of the amplifier are greater than 3 V (Figures S6A and S6C). Another point to consider is that the vibration of the woofer diaphragm causes airflow.^25^ The speed of this airflow could readily be measured by an anemometer (Figures S6B and S6D). In the frequency sweeping (1 V), only the low-frequency sound (100–300 Hz) was accompanied by a noticeable airflow. However, in the amplitude sweeping (200 Hz), in the range of 1–5 V, an airflow was observed. It is likely that both sound and airflow contribute to the deflection of droplets. The aforementioned results demonstrate that the deflection of microdroplets increases when sound frequencies are low or the amplitudes are high. It should be noted that nanodroplets and ions might also be deflected by sound or airflow, but they could not be visualized using the current optical system.

### On-line MS analyses using single woofer and four woofers

In the on-line MS experiment, the parameters that influence MS signal intensity were optimized with one large woofer, including the distance between the ESI capillary and the MS inlet (Figure S7), the drying gas flow rate (Figure S8), and the voltage applied on the ESI capillary (Figure S9). It was found that positioning the ESI capillary closer to the MS inlet led to a higher signal intensity. The drying gas flow rate was inversely proportional to the signal intensity, and, once the drying gas flow rate reached 15 L min^−1^, the strong gas flow prevented the formation of electrospray. Increasing the voltage—applied to the ESI capillary—resulted in a signal increase, but this effect became insignificant beyond 3.8 kV. However, these parameters had little effect on enhancement factors (EFs).

**Figure 4:**
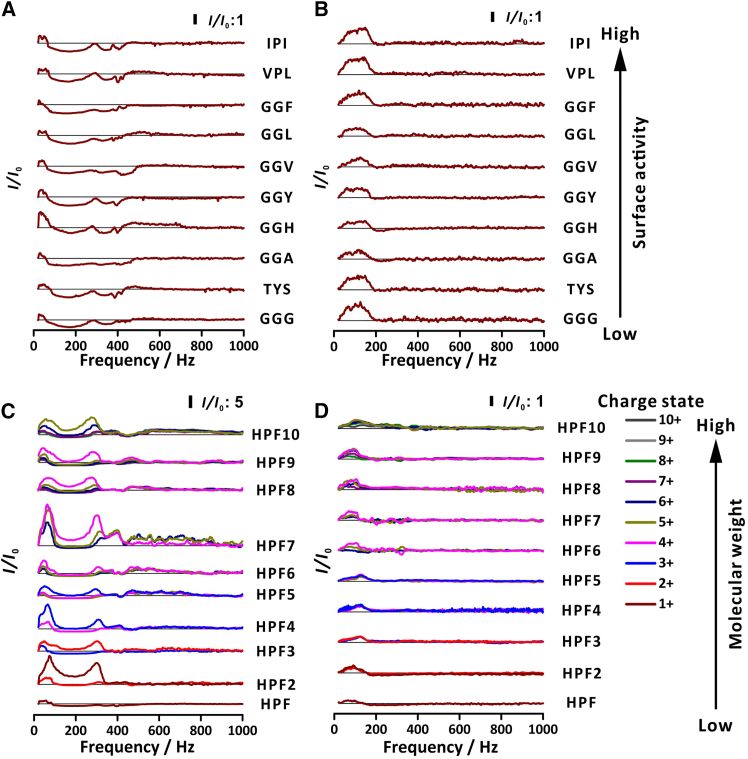
Signal enhancement of different analytes as a function of sinusoidal voltage frequency of one woofer and four small woofers (A and C) one woofer; (B and D) four small woofers. Analytes: (A and B) ten tripeptides with different surface activities; (C and D) HPF peptides with different chain lengths. Sinusoidal voltage amplitude: 1 V for the large woofer and 3 V for the four small woofers. Analytes: GGG (*m/z* 190; positive-ion mode); TYS (*m/z* 370; positive-ion mode); GGA (*m/z* 204; positive-ion mode); GGH (*m/z* 270; positive-ion mode); GGY (*m/z* 296; positive-ion mode); GGV (*m/z* 232; positive-ion mode); GGL (*m/z* 246; positive-ion mode); GGF (*m/z* 281; positive-ion mode); VPL (*m/z* 328; positive-ion mode); IPI (*m/z* 342; positive-ion mode); HPF (*m/z* 400; positive-ion mode; charge state: 1); HPFHPF (*m/z* 391, 781; positive-ion mode; charge state: 2 and 1, respectively; HPF2); HPFHPFHPF (*m/z* 388, 582; positive-ion mode; charge state: 3 and 2, respectively; HPF3); HPFHPFHPFHPF (*m/z* 387, 515; positive-ion mode; charge state: 4 and 3, respectively; HPF4); HPFHPFHPFHPFHPF (*m/z* 386, 482, 642; positive-ion mode; charge state: 5, 4, and 3, respectively; HPF5); HPFHPFHPFHPFHPFHPF (*m/z* 385, 462, 577; positive-ion mode; charge state: 6, 5, and 4, respectively; HPF6); HPFHPFHPFHPFHPFHPFHPF (*m/z* 449, 538, 673; positive-ion mode; charge state: 6, 5, and 4, respectively; HPF7); HPFHPFHPFHPFHPFHPFHPFHPF (*m/z* 439, 512, 615, 768; positive-ion mode; charge state: 7, 6, 5, and 4, respectively; HPF8); HPFHPFHPFHPFHPFHPFHPFHPFHPF (*m/z* 432, 494, 576, 691, 864; positive-ion mode; charge state: 8, 7, 6, 5, and 4, respectively; HPF9); HPFHPFHPFHPFHPFHPFHPFHPFHPFHPF (*m/z* 384, 427, 480, 548, 640, 767; positive-ion mode; charge state: 10, 9, 8, 7, 6, and 5, respectively; HPF10). The triple quadrupole mass spectrometer (QqQ-MS) was operated in selected-ion-monitoring (SIM) mode. Sample concentration: 5 μM in 25% (v/v) methanol in water. Symbols: *I*_0_, intensity without sound; *I*, intensity with sound. The horizontal black solid lines mean that *I/I*_0_ is equal to 1.

To evaluate the performance of the designed setups, we conducted experiments with twenty peptides to compare the performance with one larger woofer (Figures 4A–4C) and four small woofers setup (Figures 4B–4D). For ten tripeptides with various surface activities,^26^ the setup with four small woofers provided higher EFs, and—unlike the setup with one large woofer—it did not lead to signal decrease. For ten (HPF)_*n*_ peptides with different chain lengths, both setups provided signal enhancement.

In the setup with one large woofer, the signals of the ten tripeptides were enhanced in the frequency range of 20–80 Hz and then diminished until ∼300−400 Hz (Figure 4A). This might be because—at 20–80 Hz—the sound field affected the motion pattern of the droplets. A previous simulation showed that the droplets exposed to sound were more focused than those not exposed to sound.^21^ However, some of the peripheral droplets hit the walls of the simulation chamber under the sound field.^21^ In the ESI plume, the offspring droplets are repulsed outward.^11^ Those peripheral droplets can experience large oscillation amplitude (*cf.* Li et al.^22^ and Jia et al.^23^). Therefore, some peripheral droplets might deviate too far from the MS inlet, leading to their loss. The loss of some small droplets can lead to a reduction in space charge, thus enhancing analyte ion transmission efficiency. We hypothesized that, although the initial droplets contain the analyte, the analyte content in the droplets is different after fission processes. As the frequency was raised to the range of 100–400 Hz, the airflow speed and SPL of the large woofer were elevated to a level capable of eliminating most droplets containing the analytes (Figures S6A and S6B). Consequently, the signals were lower than those in the absence of the sound. On the other hand, above 400 Hz, the strength of sound was insufficient to influence the droplets, and the accompanying airflow almost ceased. Thus, the signal gradually returned to the same level as if no sound had been applied.

When using the setup with four small woofers, we observed signal enhancement in the frequency range of 20–200 Hz (Figure 4B). Because the sound beams—generated by the four small woofers arranged symmetrically—converged and collimated the electrospray plume, more analyte-containing droplets entered the MS inlet. Since the high-frequency sound was not strong enough to affect the droplets, the signal returned to the same level as if the sound was turned off. In addition, because the sound beams of the four symmetrically arranged small woofers compensated for each other—and kept the electrospray plume from deflecting—no signal drop was seen—unlike in the setup with one large woofer. All of the various surface-active tripeptides exhibited similar trends when using one large woofer and four small woofers. We attributed this to the similarity in sequences and the number of amino acid residues. It is likely that the ten tripeptides tended to stay in the droplets of similar size.

Among the ten HPF peptides with varying numbers of repeating units, we observed signal enhancement in the low-frequency region in both setups (Figures 4C and 4D). However, these enhancements differed in terms of frequency ranges, patterns, and values. In the setup with one large woofer, signal enhancement resulted from the oscillatory motion of analyte-laden droplets within the sound field, which concentrated them more closely toward the MS inlet, while smaller droplets were removed by sound and airflow. This also led to a reduction in space charge and improvement in transmission efficiency. The decrease in EF—observed at ∼200 Hz—can be attributed to the airflow accompanying the sound. In the setup with four small woofers, signal enhancement resulted from the sounds converging and collimating the electrospray plume (Figure 4D). The disparity in the frequency range of signal enhancement between the two setups stems from the differences in woofer power and geometry. The large woofer has greater power (30 W) than the small woofers (each 7 W). The greater the sound pressure, the more significant is the impact on the droplets.^22^^,^^23^ In addition, differences in the woofer geometries also led to differences in the spatial distribution of sound pressure. The single large woofer demonstrated better performance with long-chain peptides because these peptides tended to reside in larger droplets, which were not effectively removed by the unidirectional sound beam. This might be due to the large mass of long-chain peptides, which results in lower mobility compared to short-chain peptides. When a large droplet undergoes fission, smaller molecules preferentially migrate to the jet due to electrophoresis and diffusion, while larger molecules are left behind in the bigger droplet. However, droplets containing these peptides might be too large to be focused well using the setup with four small woofers. Moreover, in the large woofer setup, low-charged peptide ions exhibited higher EFs compared to highly charged peptide ions. This could be attributed to the droplets being subjected to shear forces as they fly through dense gas, causing droplets to undergo deformation.^27^ Some analytes may be closer to the surface of the droplet, making them easier to acquire surface charges. Besides, small progeny droplets—with high surface charge densities—are rich in surface-active species after asymmetric fission process.^28^ Consequently, one can speculate that highly charged peptide ions are mainly derived from small droplets, while low-charged peptide ions originate from large droplets. However, in the setup with four small woofers, the ESI plume was collimated, resulting in no significant difference in the EFs between highly charged peptide ions and low-charged peptide ions.

Quantitative performances of the setups with one large woofer and four small woofers have been evaluated using GGY and HPF7 peptides. The large woofer was supplied with the signal with frequency of 35 Hz and amplitude 1 V, while the four small woofers were supplied with the signals with frequency 100 Hz and amplitude 3 V, which correspond to the local maxima in Figure 4. The calibration plots are presented in Figure S10, and the related equations are shown in Tables S1 and S2. It is pleasing to note that the calibration datasets are linear in the range 1–20 μM. The slopes with sound are greater than the slopes without sound. The coefficients of determinations (*R*^2^) are above 0.99 for all the datasets obtained with sound. The limits of detection (LODs) are lower with sound than without sound. In the case of HPF7—analyzed with one large woofer—the difference is over 5-fold.

To further explore the benefits of the proposed method (using one large woofer) in MS analysis, we have analyzed 5 μM HPF7 (in 25% [v/v] aqueous methanol) 10 times in one day. We focused on the *m/z* 673 (charge state: +4). At 35 Hz, the EF was 3.59 ± 0.67 (relative standard deviation [RSD], 18.7%), which is deemed satisfactory considering that the prototype device was used. Further, we calculated the signal-to-noise (*S/N*) ratios (with sound vs. without sound), and the value was 13.3 ± 0.4 (*S*, average of signal over 30 s; *N*, standard deviation of blank signal over 30 s). Thus, it is pleasing to note that the proposed method helps to reduce interference by increasing *S/N* ratios. The same sample was analyzed in 5 days, and the inter-day RSD of the EFs was 23.6%. Further, we explored the possibility to reduce interference of real matrices. For that purpose, we obtained liquid from a cosmetic facial mask and diluted it 1,000× with 25% (v/v) aqueous methanol and 0.1% formic acid. We observed enhancements of unidentified peaks at the *m/z* 137, 181, and 223 at 35 Hz (Figure S11A). Then, we spiked the same sample matrix with 5 μM HPF7 and 5 μM lysine and also observed signal enhancements (Figures S11B and S11C). This result shows that the method performs well even in the presence of a complex matrix and without chromatographic separation.

**Figure 5:**
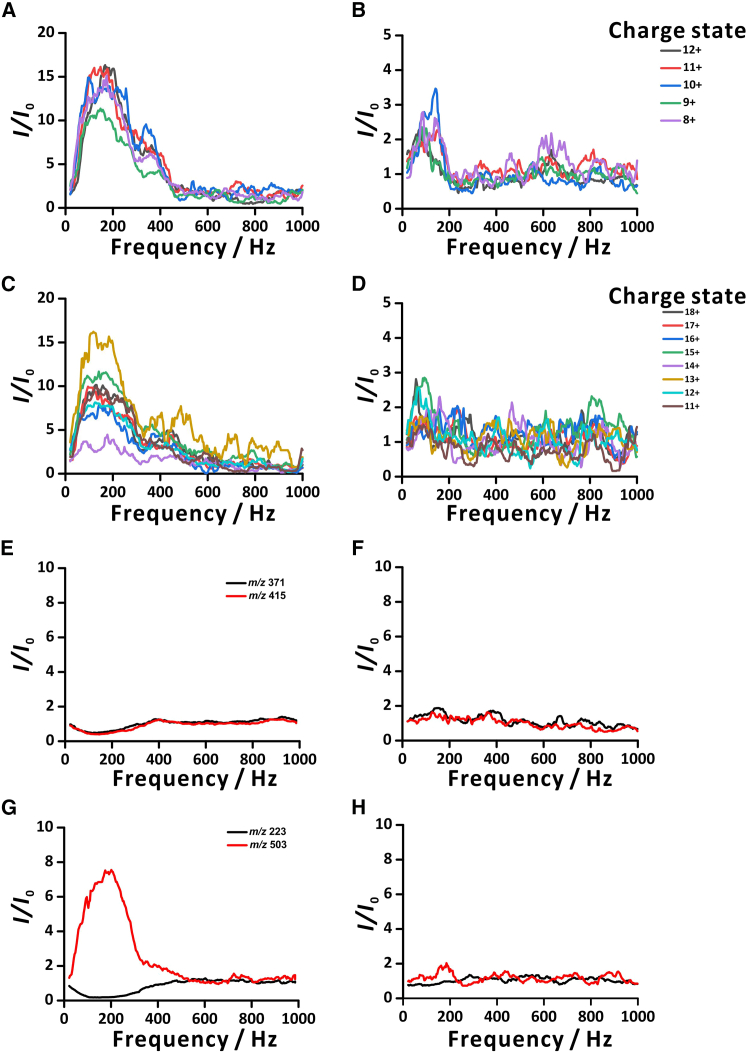
Signal enhancement of different analytes as a function of sinusoidal voltage frequency of one woofer and four small woofers (A, C, E, and G) one woofer; (B, D, F, and H) four small woofers. Analytes: (A and B) ubiquitin; (C and D) cytochrome *c*; (E and F) PEG 400; (G and H) PEG 1000. Sinusoidal voltage amplitude: 1 V for the large woofer and 3 V for the four small woofers. The triple quadrupole mass spectrometer (QqQ-MS) was operated in Q3 mode. For proteins, sample concentration: 5 μM in 25% (v/v) methanol in water containing 10 mM ammonium acetate and 0.1% (v/v) formic acid. For PEG polymers, sample concentration: 0.05 μg μL^−1^ in 25% (v/v) methanol in water containing 0.1% (v/v) formic acid. Symbols: *I*_0_, intensity without sound; *I*, intensity with sound.

Furthermore, we conducted experiments with polymers—including proteins and synthetic polymers—using the setups with one larger woofer (Figures 5A–5C, 5E, and 5G) and four small woofers (Figures 5B–5D, 5F, and 5H). The corresponding polymer spectra are presented in Figures S12 and S13. For the proteins, we could observe that the setup with one large woofer yielded higher *EF*s than the setup with four small woofers (Figures 5A–5D). In the setup with one large woofer, intensities of both ubiquitin and cytochrome *c* were enhanced up to ∼500 Hz (Figures 5A–5C). In the setup with four small woofers, the intensity of ubiquitin was enhanced up to ∼300 Hz, while the intensity of cytochrome *c* did not show a significant increase (Figures 5B–5D). This might be due to the large mass of proteins, which results in low mobility and causes the molecules to be left behind in the bigger droplets. Consequently, the performance in the setup with one large woofer was better than that in the setup with four small woofers. The mass of cytochrome *c* (∼12,348 Da) is larger than that of ubiquitin (∼8,564 Da). Thus, in the setup with four small woofers, the enhancement of ubiquitin was greater than that of cytochrome *c*. As for the average charge states of proteins (Figure S14), there were no significant changes in the both setups. For polyethylene glycol (PEG), intensities of PEG 400 were decreased in the setup with one large woofer (Figure 5E). However, for PEG 1000, we found that the intensity of the low *m/z* (*m/z* 223) signal dropped, while the intensity of the high *m/z* (*m/z* 503) signal rose (Figure 5G). In the setup with four small woofers, the intensities of PEG 400 and PEG 1000 had no significant difference (Figures 5F–5H). The observed signal decreases may be because the analyte species were concentrated in small droplets, and such droplets were selectively removed in the setup with one large woofer. On the other hand, the small woofers collimated the electrospray plume to prevent the signal from decreasing. The small droplets are located in the peripheral zone of the plume. When the droplets in the peripheral zone are focused by the four small woofers, they are also repelled by larger droplets in the central zone. This repulsion could explain why the signal increase was only moderate in that case.

### Further verification of analyte partitioning to droplets of different sizes

To demonstrate that different analytes partition to droplets with different sizes, we performed a simple test using an amino acid mixture and a tripeptide mixture. We shifted the ESI capillary toward the left hand side away from the inlet by a certain offset distance (Figure S15A). We found that the intensity ratio of phenylalanine and total ion currents (TICs; the sum of the individual extracted ion currents [EICs] of alanine, lysine, and phenylalanine) increased as the offset distance increased, but the intensity ratio of alanine and TIC, and the intensity ratio of lysine and TIC, decreased as the offset distance increased (Figure S15B). When the ESI capillary was moved to the left, the intensity ratio of lysine and TIC decreased before the intensity ratio of alanine and TIC started to decrease. It was previously shown that the smaller satellite droplets—produced during asymmetric fission processes—are rich in compounds with high surface activity.^28^ Phenylalanine is a highly hydrophobic amino acid, so phenylalanine should exhibit a higher surface concentration than the hydrophilic amino acids.^26^^,^^29^ As expected, phenylalanine remained in the peripheries of the plume, alanine occupied the sub-peripheral zone, and lysine resided in the center. In fact, it is known that space-charge effect causes the offspring droplets to be repelled outward.^11^ The droplets in the center are larger than the ones in the periphery.^11^

We also performed a similar experiment with three tripeptides with different surface activities: IPI > GGY > GGG (*cf.* Cech et al.^26^). For the peptide mixture, we observed that the intensity ratio of IPI and TIC (the sum of the individual EICs of IPI, GGY, and GGG) increased with the offset distance, while the intensity ratio of GGY and TIC, and the intensity ratio of GGG and TIC, decreased as the offset distance increased (Figure S15B). When the ESI capillary was shifted to the left, the intensity ratio of GGG and TIC decreased before the intensity ratio of GGY and TIC began to decrease. It can be concluded that IPI remained in the droplets in the peripheral zone of the plume, while GGY occupied the sub-peripheral zone of the plume, and GGG resided in the central zone of the plume. This experiment shows that analytes with different properties really partition to droplets of different sizes. Thus, the aforementioned explanation—concerning the effect of sound beam on electrospray plume—is viable.

### Considerations regarding the acoustic noise

The use of sound to enhance performances of laboratory procedures raises questions about safety and convenience of the method. Long-term exposure to noise levels above 75 dB can impair the ability to hear sounds and understand their meaning.^30^ Additionally, noise is a stressor that affects the autonomic nervous system and endocrine system, potentially leading to hypertension and ischemic heart disease.^31^^,^^32^ We measured sound intensity at a distance of ∼1 m from the mass spectrometer with the speaker off and on, and the values were 61.0 ± 0.4 and 75.6 ± 0.3 dBA, respectively. Therefore, it is recommended to use a soundproof box or wear protective earmuffs to mitigate the negative impact of sound on the human body when using these setups.

### Concluding remarks

Using high-speed imaging, we have verified the possibility to selectively deflect small microdroplets present in electrospray plume with a beam of low-frequency sound. We have further shown the possibility to modulate MS signal in on-axis electrospray MS geometry. We have demonstrated two methods to manipulate the electrospray plume. In the setup with one large woofer, we utilized a unidirectional sound beam to deflect the electrospray plume and selectively remove smaller droplets, improving transmission efficiency of large analyte-containing droplets. In the setup incorporating four small woofers, four sound beams converged to shape the electrospray plume, making more analyte-containing droplets enter the MS inlet. We suggest that the setup with four small woofers enables transmission of a larger proportion of small droplets, which normally occupy peripheries of electrospray plume by counteracting space-charge effect. In perspective, the efficiency of plume focusing can further be enhanced by incorporating a larger number of miniature woofers around the plume circumference. It is suggested that the study findings will not only support the development of improved analytical methods based on MS but also assist other procedures involving electrospray, for example, synthesis of nanomaterials.

### Limitations of the study

While our findings provide a way to modulate electrospray process, potentially leading to useful applications, we would like to acknowledge two limitations of the approach. First, the high-speed imaging method can only visualize microdroplets and is blind to nanodroplets, which are important for ion formation in ESI. Second, the use of low-frequency sound may be inconvenient for some users of the proposed technique unless appropriate precautions are taken.

## Resource availability

### Lead contact

Requests for further information and resources should be directed to and will be fulfilled by the lead contact, Pawel L. Urban (urban@mx.nthu.edu.tw).

### Materials availability

This study did not generate new unique reagents.

### Data and code availability

• The data reported in this paper will be shared by the lead contact upon reasonable request.
• The original code is available in this paper’s supplemental information.
• Any additional information required to reanalyze the data reported in this paper is available from the lead contact upon request.

## Acknowledgments

We acknowledge the 10.13039/100020595National Science and Technology Council, Taiwan (grant numbers 112-2113-M-007-025-MY2 and 110-2628-M-007-004-MY4). We also thank Krzysztof Buchowiecki for assistance.

## Author contributions

Conceptualization, P.L.U.; methodology, Y.-H.C. and P.L.U.; software, Y.-H.C.; formal analysis, Y.-H.C.; investigation, Y.-H.C. and M.-M.H.; resources, P.L.U.; data curation, Y.-H.C. and M.-M.H.; writing – original draft, Y.-H.C.; writing – review and editing, M.-M.H. and P.L.U.; visualization, Y.-H.C.; supervision, P.L.U.; project administration, P.L.U.; funding acquisition, P.L.U.

## Declaration of interests

The authors declare no competing interests.

## STAR★Methods

### Key resources table

**Table:** 

REAGENT or RESOURCE	SOURCE	IDENTIFIER
**Chemicals, peptides, and recombinant proteins**		

Methanol (for LC-MS)	Merck	cat. no. 1.06018.1000
Water (for chromatography, LiChrosolv)	Merck	cat. no. 115333
Ammonium acetate (98+%, for HPLC)	Acros Organics	cat. no. 219992500
L-lysine (≥98%, for TLC)	Sigma-Aldrich	cat. no. L5501
L-phenylalanine (98.5–101.1%)	Sigma-Aldrich	cat. no. P5482
L-alanine (99%)	Acros Organics	cat. no. 102830250
Cytochrome c (90%, from horse heart muscle)	Acros Organics	cat. no. 147531000
Formic acid (>98%)	Thermo Fisher Scientific	cat. no. 147932500
Ubiquitin (human recombinant)	Boston Biochem	cat. no. U-100H
Polyethylene glycol 400	Alfa Aesar	cat. no. B21992
Polyethylene glycol 1000	Showa	cat. no. 1624-4350
GGG	BioAb	cat. no. 1307
GGA	BioAb	cat. no. 1305
GGV	BioAb	cat. no. 1302
GGL	BioAb	cat. no. 1301
GGY	BioAb	cat. no. 1303
GGF	BioAb	cat. no. 1300
VPL	BioAb	cat. no. 1299
GGH	BioAb	cat. no. 1304
IPI	BioAb	cat. no. 1298
TYS	BioAb	cat. no. 1306
HPF	BioAb	cat. no. 252657
HPFHPF	BioAb	cat. no. 926121
HPFHPFHPF	BioAb	cat. no. 926122
HPFHPFHPFHPF	BioAb	cat. no. 926123
HPFHPFHPFHPFHPF	BioAb	cat. no. 926124
HPFHPFHPFHPFHPFHPF	BioAb	cat. no. 1052415
HPFHPFHPFHPFHPFHPFHPF	BioAb	cat. no. 1052416
HPFHPFHPFHPFHPFHPFHPFHPF	BioAb	cat. no. 1052417
HPFHPFHPFHPFHPFHPFHPFHPFHPF	BioAb	cat. no. 1052418
HPFHPFHPFHPFHPFHPFHPFHPFHPFHPF	BioAb	cat. no. 1052419

**Deposited data**		

Main mass spectrometry data	Figshare	https://figshare.com/articles/dataset/Shaping_Electrospray_Plume_with_Convergent_Sound_Beams/28705445?file=53366165

**Software and algorithms**		

WaveForms software	Digilent	version WF 3.18.1
Python	Python Software Foundation	version 3.10
ImageJ	National Institutes of Health	version 1.54h
Matlab	MathWorks	version R2023a
LabSolutions	Shimadzu	version 5.97
Excel	Microsoft	version 2019
OriginPro	OriginLab	version 8.5

**Other**		

DC-DC converters	Centenary Materials Company	part no. LM2596S
Amplifier (D class; gain value: 11)	Centenary Materials Company	part no. SL-AA50SD
Relay board	Centenary Materials Company	part no. 119307
Woofer with a plastic funnel (diameter, 4 inch, power, 30 W, nominal frequency range, 65 Hz – 5 kHz, impedance, 4 Ω, sensitivity, 86 ± 3 dB)	JOJaudio	part no. 137CR
Function generator (Analog Discovery 2) with a BNC adapter board	Digilent	part no. 210-321
DC-DC converters	Golden Electrics	part no. LM2596
Amplifiers (D class; supply DC voltage, 12 V; power, 50 W)	Taiwan Intelligent Sensor Technology	part no. TPA3116D2
Woofers (side length, 53.5 mm; power, 7 W; impedance, 4 Ω; sensitivity, 84 ± 2 dB)	In Hann	part no. 53S-IN
Custom-made funnel-shaped collimators (material, stainless steel)	Lilong Steel	N/A
Customized holder (material, aluminum)	NTHU workshop	N/A
ESI capillary (I.D., 100 μm; O.D., 270 μm; length, 82.5 mm)	Shimadzu	part. no 225-14915
Acrylonitrile-butadiene-styrene filament	Tiertime	part. no C-21-02
Power supply	Spellman	part no. MPS10P10
Peristaltic pump	Yotec Precision Instrument	part no. MF-10
Tygon tubing (length: 381 mm; O.D.: 0.89 mm; I.D.: 0.25 mm)	Pulse Instrumentation	part no. 28453037
PTFE tubing (length: 100 mm; O.D.: 1.58 mm; I.D.: 0.30 mm)	Merck	part no. 58702
Metal union	IDEX Health & Science	part no. U-438
PTFE tubing (length: 400 mm; O.D.: 1.58 mm; I.D.: 0.30 mm)	Merck	part no. 58702
Machine-vision high-speed camera	Phantom	part no. S710
Stereomicroscope	Nikon	part no. SMZ745T
Fiber illuminator	Thorlabs	part no. OSL2IR
Frame grabbers	Euresys	model Coaxlink Octo
Cables	Components Express	part no. CX-34-1-34-05 CoaXPress Cables
Computer	Asus	model W980T workstation
Function generator	Twintex	part no. TFG-3605E
External I/O interface	Euresys	part no. HD26F 3304 external I/O connector
Triple quadrupole mass spectrometer	Shimadzu	model LCMS-8030

### Method details

#### Electronic circuit for one large woofer

An AC-DC adapter (input, 110 V; output, 19 V; current, 3.42 A) was connected to two DC-DC converters (model no. LM2596S) to supply 12 V to the amplifier (D class; gain value: 11) and 5 V to the relay board. The woofer with a plastic funnel (diameter, 4 inch, power, 30 W, nominal frequency range, 65 Hz – 5 kHz, impedance, 4 Ω, sensitivity, 86 ± 3 dB) was connected to the left channel of the amplifier. The amplifier was connected to a waveform channel 1 of a function generator (Analog Discovery 2) with a BNC adapter board. The Analog Discovery 2 module was operated using the WaveForms software to generate sine waveforms with the specific frequency and amplitude. The relay board (input pin, IN1) was connected to pin 7 of the Analog Discovery 2 module to trigger the mass spectrometer for on-line data acquisition.

#### Electronic circuit for four small woofers

An AC-DC adapter (input, 110 V; output, 19 V; current, 3.42 A) was connected to four DC-DC converters (model no. LM2596) to supply 12 V to four amplifiers (D class; supply DC voltage, 12 V, power, 50 W). Four woofers (side length, 53.5 mm; power, 7 W; impedance, 4 Ω; sensitivity, 84 ± 2 dB) with custom-made funnel-shaped collimators (material, stainless steel) were connected to the left channels of the amplifiers. The four woofers were placed on the customized holder (material, aluminum). The four amplifiers were connected to respective waveform generator channels of two Analog Discovery 2 with BNC adapter boards. Two Analog Discovery 2 modules were operated using a program written in Python to generate sine waveforms with the specific frequency and amplitude (see the supplemental information).

#### Off-line electrospray setup

An ESI capillary was installed in the 3D-printed holder (core material, acrylonitrile-butadiene-styrene). A high-voltage (HV) power supply was connected to the ESI capillary to generate electrospray. The sample was pumped by a peristaltic pump, transferred through a section of Tygon tubing (length: 381 mm; O.D.: 0.89 mm; I.D.: 0.25 mm), a section of polytetrafluoroethylene (PTFE) tubing (length: 100 mm; O.D.: 1.58 mm; I.D.: 0.30 mm), via a grounded metal union, and another section of PTFE tubing (length: 400 mm; O.D.: 1.58 mm; I.D.: 0.30 mm) to the ESI capillary.

#### Off-line high-speed camera setup

For the purpose of capturing the images of electrospray microdroplets, a machine-vision high-speed camera was coupled with a stereomicroscope. The resolution of the high-speed camera was set to 1024 × 640. The frame rate was set to 10,000 fps. The exposure time was set to 1 μs. A fiber illuminator was placed in front of a stereomicroscope as a light source. The ESI capillary was positioned perpendicularly to a counter-electrode (60 mm × 60 mm; thickness: 0.8 mm; material: glass fiber with copper coated on one side) in an upside-down configuration with ESI capillary facing downward and a counter-electrode facing upward. The woofer was positioned perpendicularly to the axis of the ESI capillary and the counter-electrode. The distance between the ESI capillary tip and the counter-electrode was ∼ 8 mm. The distance between the funnel of the woofer and the ESI capillary axis was 55 mm. A liquid sample [25% (v/v) methanol in water] was delivered at a flow rate of ∼50 μL min^–1^ by the peristaltic pump. The HV power supply provided 3.8 kV to the ESI capillary. The ESI capillary, counter-electrode, and the woofer were placed in the middle between the stereomicroscope and the light source. The high-speed camera was linked to two frame grabbers through 16 cables. The frame grabbers were inserted to the PCIe3 interface of a computer. The channel A of the function generator (TFG-3605E) was connected to pin 12 of an external I/O interface of the frame grabber. We used the function generator to produce square waveforms (V_pp_, 5 V; frequency, 10 kHz; duty cycle, 50%). When the external I/O interface received the square waveforms, the high-speed camera was triggered to capture the images.

#### On-line ESI-MS setup

A triple quadrupole mass spectrometer was employed to detect ions. The ESI source of the mass spectrometer was replaced with the off-line electrospray setup. The distance between the ESI capillary tip and the MS inlet was 8 mm. The distance between the funnel of the large woofer and the ESI capillary axis was 55 mm. In the setup incorporating four small woofers, the distance between the ESI capillary tip and the MS inlet was 8 mm. The distance between the plane where the four funnel tips were located and the MS inlet was 10 mm. A liquid sample was delivered at a flow rate of ∼ 50 μL min^–1^. The sample was delivered to the ESI capillary in the same way as in the off-line electrospray setup. The HV power supply provided 3.8 kV to the ESI capillary. The mass analyzer was operated in the positive-ion SIM mode for amino acids and peptides and in Q3 scan mode for proteins and polymers. The flow rate of drying gas (nitrogen) was 3.0 L min^–1^; the temperature of the heat block was 400°C; the temperature of the desolvation line was 250°C.

### Quantification and statistical analysis

#### Image processing

The raw images were processed in bulk using a program written in Python. Initially, the raw images in BMP image format were smoothed using the median filter function (kernel size, 9). Subsequently, the images were processed using an adaptive Gaussian thresholding algorithm (*cv2.adaptiveThreshold*; Gaussian window block size, 51; constant, 3). Finally, the processed images underwent conversion into binary images, and were saved in JPG format files. After processing, ImageJ was utilized to analyze individual droplets within the images in bulk based on the customized script in ImageJ Macro. First, the scale bar was set to ascertain the size of droplets. Then, a pixel intensity threshold was selected to distinguish the droplets from the background. The droplet sizes were measured by the “Analyze Particles” function (size: 100-12500 μm^2^; circularity: 0.30-1.00). Ultimately, the size and center of each individual droplet shadow were recorded. The results—obtained from processing all the images—were saved in CSV files. The CSV files were then imported to Matlab to calculate diameters and distances from the center of the ESI capillary.

#### MS data analysis

The raw data of EICs for selected ions and—in some cases—TICs were exported as ASCII files from the LabSolutions software. These files underwent processing through the customized script in Matlab. First, the data underwent processing using a median filter (20-point window). Subsequently, the ion intensity without sound (*I*_0_), the ion intensity with sound (*I*), and the *EF*s were computed:

(Equation 1) $$EF=I/I_{0}$$

Following this, the processed data were imported into Excel, where the three replicates were averaged to generate *EF* plots. The datasets were then visualized using OriginPro.
