# Supplementary material for: Shaping electrospray plume with convergent sound beams
Source: iScience. 2025 Apr 8;28(5):112388. doi: 10.1016/j.isci.2025.112388 (PMC12245440; doi:10.1016/j.isci.2025.112388)
Supplement: Document S1. Figures S1–S15, Tables S1 and S2, and Methods S1 [file mmc1.pdf]

iScience, Volume *28*

Supplemental information

Shaping electrospray plume
with convergent sound beams

Yu-Hao Chen, Min-Min Hung, and Pawel L. Urban

ADDITIONAL TABLES

Table S1. Calibration equations and LODs obtained with the setup incorporating one large woofer. Sinusoidal voltage amplitude: 1 V. Analytes: GGY (*m/z* 296; positive-ion mode; charge state: 1); HPF7 (*m/z* 673; positive-ion mode; charge state: 4). The QqQ-MS was operated in SIM mode. Sample concentration: 1, 5, 10, 15, 20 µM in 25% (v/v) methanol in water containing 0.1% (v/v) formic acid. $LODs = \frac{3.3\sigma}{m}$. Symbols: I − intensity; c − concentration (µM); σ − standard deviations (errors) of the intercepts of the calibration equations; m − slopes of the calibration equations.

Compounds	Frequency	Calibration equations	R^2	LODs
GGY	0 Hz	$I = (35319 \pm 1564) \times c + (72289 \pm 19925)$	0.9922	1.86 µM
	35 Hz	$I = (84246 \pm 3512) \times c + (35319 \pm 27624)$	0.9931	1.08 µM
HPF7	0 Hz	$I = (48118 \pm 10275) \times c + (-26655 \pm 15123)$	0.8396	1.04 µM
	35 Hz	$I = (213868 \pm 10565) \times c + (35508 \pm 11559)$	0.9903	0.18 µM

Table S2. Calibration equations and LODs obtained with the setup incorporating four small woofers. Sinusoidal voltage amplitude: 3 V. Analytes: GGY (*m/z* 296; positive-ion mode; charge state: 1); HPF7 (*m/z* 673; positive-ion mode; charge state: 4). The QqQ-MS was operated in SIM mode. Sample concentration: 1, 5, 10, 15, 20 µM in 25% (v/v) methanol in water containing 0.1% (v/v) formic acid. $LODs = \frac{3.3\sigma}{m}$. Symbols: I − intensity; c − concentration (µM); σ − standard deviations (errors) of the intercepts of the calibration equations; m − slopes of the calibration equations.

Compounds	Frequency	Calibration equations	R^2	LODs
GGY	0 Hz	$I = (49716 \pm 3741) \times c + (44813 \pm 27307)$	0.9833	1.81 µM
	100 Hz	$I = (82188 \pm 3615) \times c + (111087 \pm 42948)$	0.9923	1.72 µM
HPF7	0 Hz	$I = (30805 \pm 7945) \times c + (-21964 \pm 14485)$	0.7782	1.55 µM
	100 Hz	$I = (90573 \pm 4709) \times c + (-58715 \pm 18321)$	0.9893	0.67 µM

ADDITIONAL FIGURES

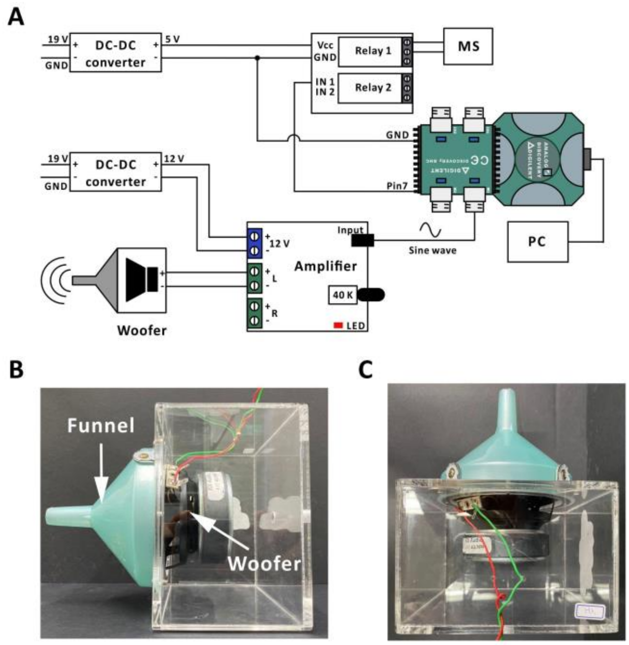

Figure S1. Setup with large woofer: (A) schematic of the large woofer circuit; (B) side view of the large woofer, and (C) top view of the large woofer.

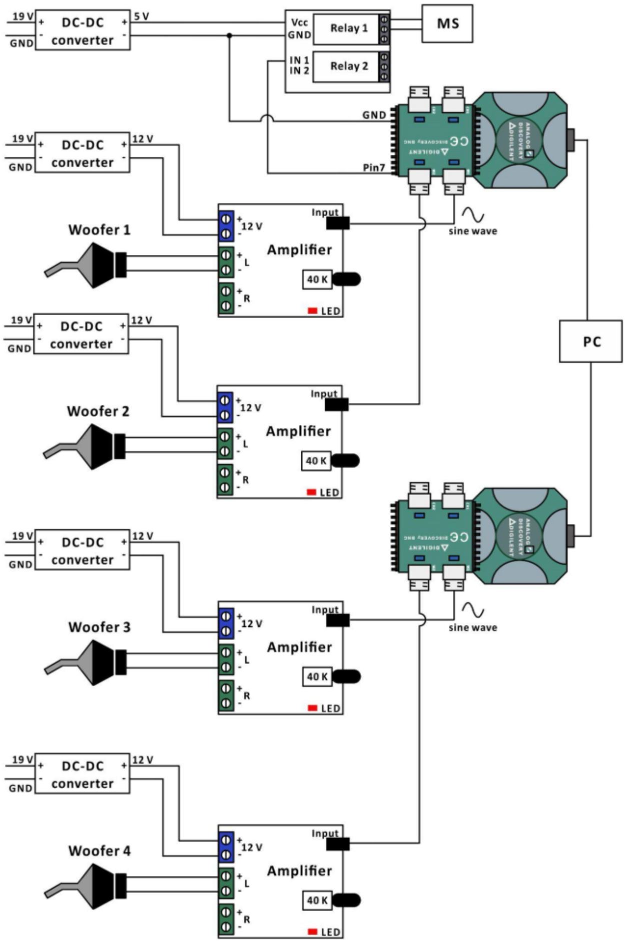

Figure S2. Schematic of the small woofers circuit.

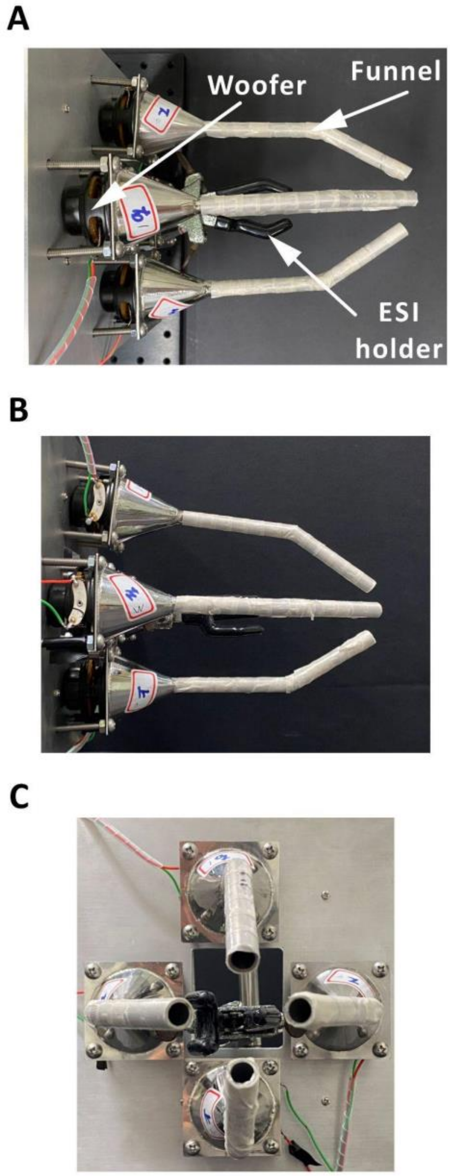

Figure S3. Photos of the small woofers: (A) top view of the small woofers; (B) side view of the small woofers, and (C) front view of the small woofers.

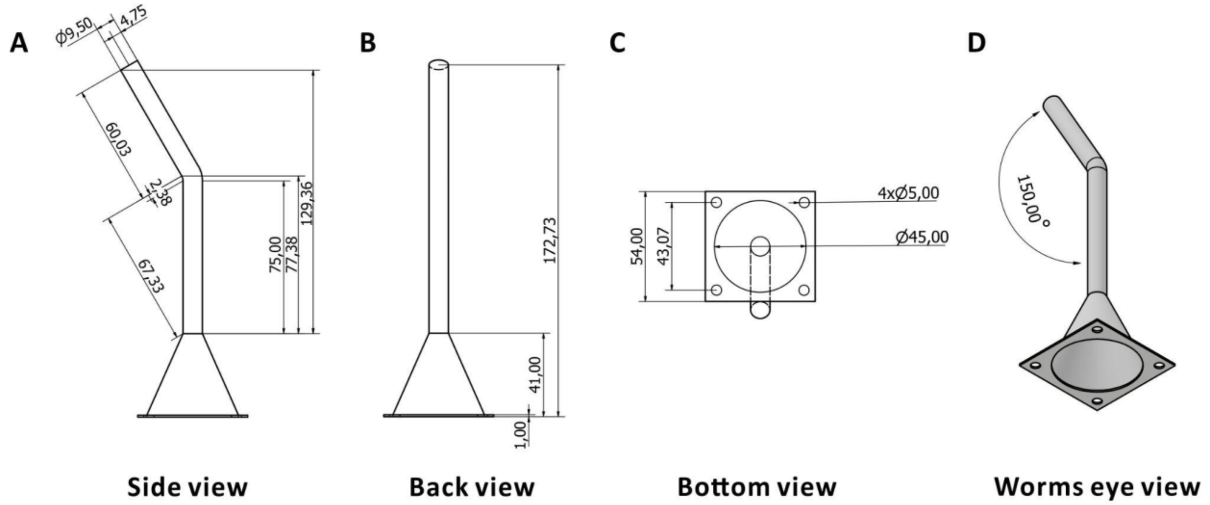

Figure S4. Technical drawing of the small woofer funnel: (A) side view of small woofer funnel; (B) back view of small woofer funnel; (C) bottom view of small woofer funnel; (D) worm's-eye view of small woofer funnel.

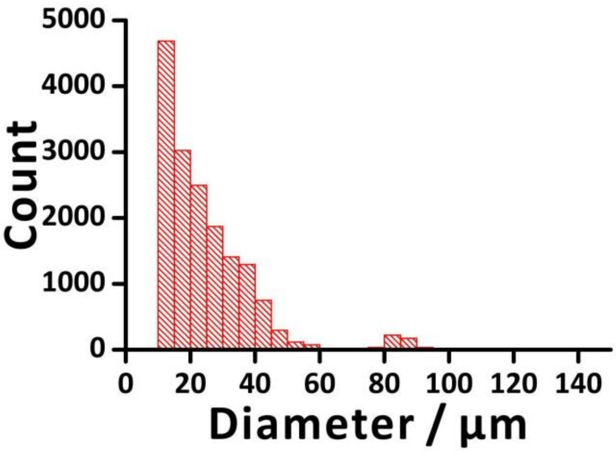

Figure S5. Histogram of microdroplets diameters in the condition without sound. Sample: 25% (v/v) methanol in water. Voltage applied to the ESI capillary was 3.8 kV. The flow rate was 50 µL min^{-1}. The number of images was 900.

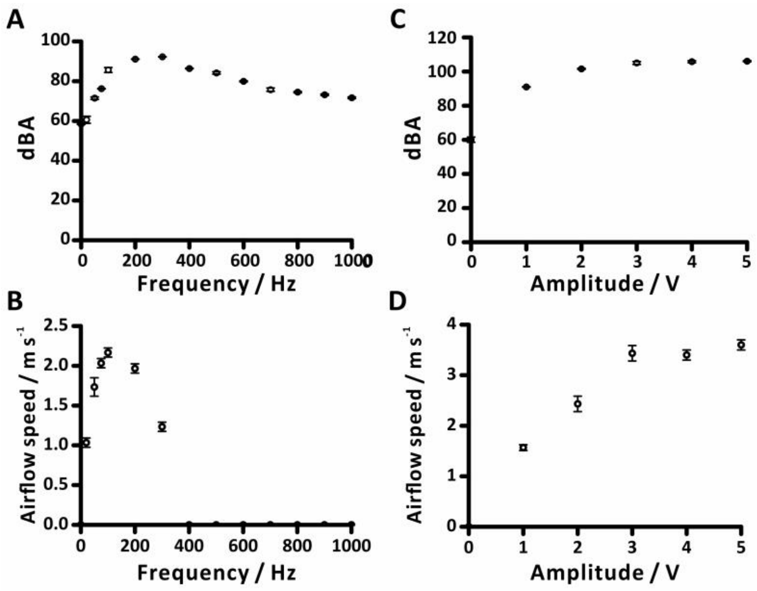

Figure S6. Measurement of sound pressure level and airflow speed of the larger woofer: (A) sound pressure levels with various frequencies; (B) airflow speeds with various frequencies; (C) sound pressure levels with various amplitudes; (D) airflow speeds with various amplitudes. Default sinusoidal voltage amplitude: 1 V. Default sinusoidal voltage frequency: 200 Hz. The distance between the larger woofer and anemometer or decibel meter was 8 mm. Replicates, n = 3. Data are represented as mean ± SD.

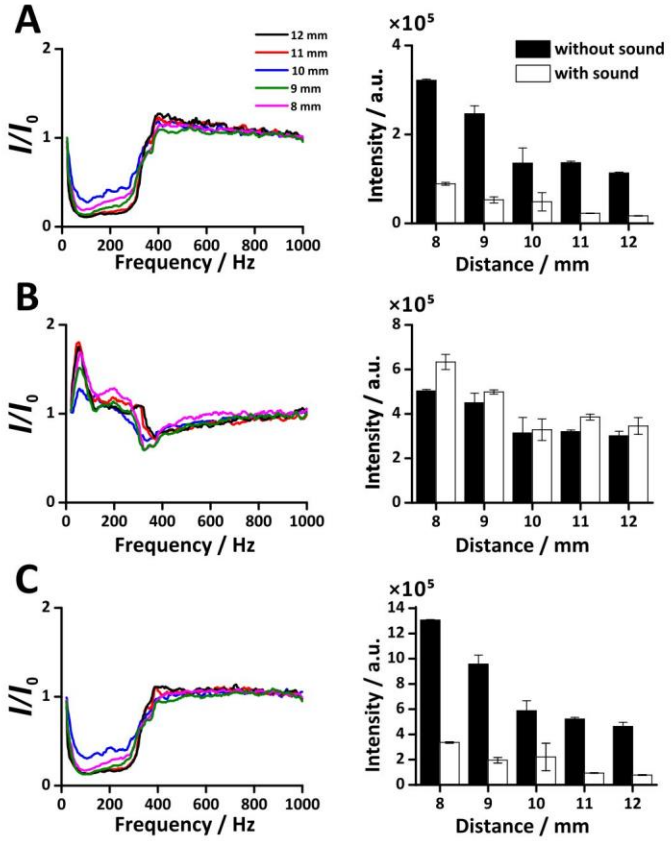

Figure S7. Influence of the distance between ESI capillary and MS inlet on *EF* and intensity in the presence of sound. (A) *EF* (left) and intensity (right) for alanine (*m/z* 90; positive-ion mode) as a function of sinusoidal voltage frequency at different distances. (B) *EF* (left) and intensity (right) for lysine (*m/z* 147; positive-ion mode) as a function of sinusoidal voltage frequency at different distances. (C) *EF* (left) and intensity (right) for phenylalanine (*m/z* 166; positive-ion mode) as a function of sinusoidal voltage frequency at different distances. Sinusoidal voltage amplitude: 1 V. Sample concentration: 5 µM in 25% (v/v) methanol in water. Sample was injected into the ESI source by a peristaltic pump at a flow rate of 50 µL min^{-1}. The three replicates were averaged to generate *EF* plots. Intensities obtained with sound from 50 to 300 Hz were averaged, and shown in the bar plots. The number of averaged values was 248. Symbols: I_0 − intensity without sound; I − intensity with sound. Data are represented as mean ± SD.

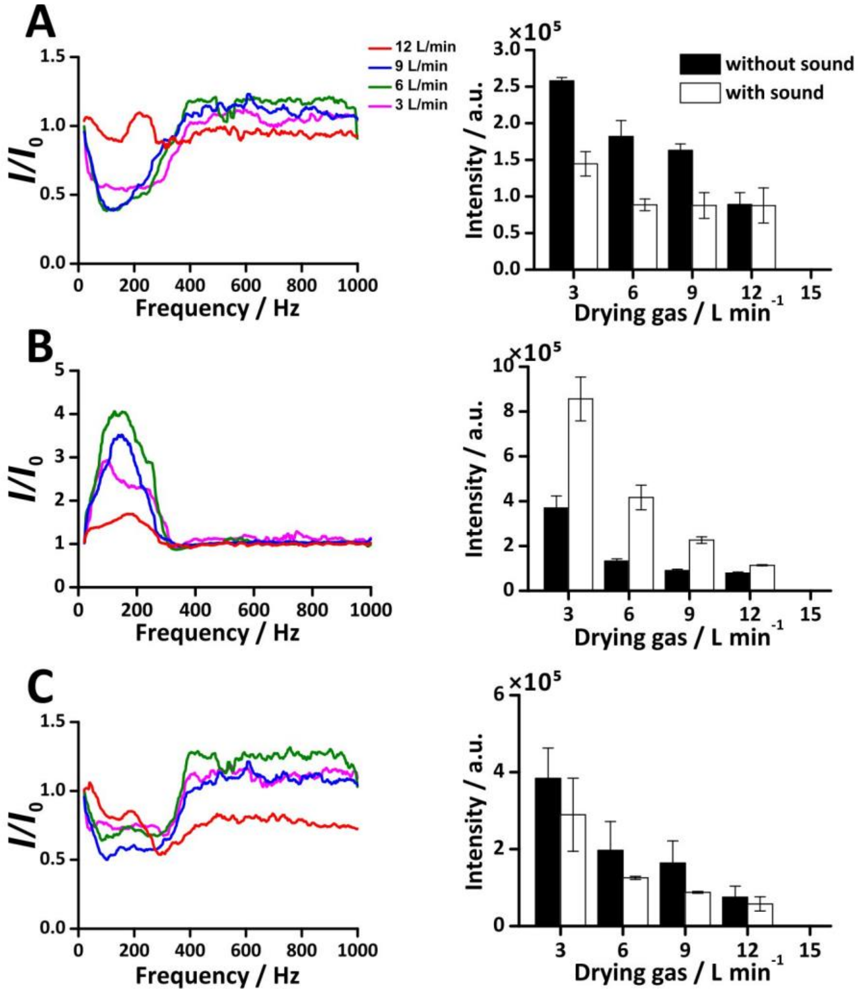

Figure S8. Influence of the flow rate of drying gas on *EF* and intensity in the presence of sound. (A) *EF* (left) and intensity (right) for alanine (*m/z* 90; positive-ion mode) as a function of sinusoidal voltage frequency at different distances. (B) *EF* (left) and intensity (right) for lysine (*m/z* 147; positive-ion mode) as a function of sinusoidal voltage frequency at different distances. (C) *EF* (left) and intensity (right) for phenylalanine (*m/z* 166; positive-ion mode) as a function of sinusoidal voltage frequency at different distances. Sinusoidal voltage amplitude: 1 V. Sample concentration: 5 μM in 25% (v/v) methanol in water. Sample was injected into the ESI source by a peristaltic pump at a flow rate of 50 μL min^{-1}. The three replicates were averaged to generate *EF* plots. Intensities obtained with sound from 50 to 300 Hz were averaged, and shown in the bar plots. The number of averaged values was 248. Symbols: I_0 − intensity without sound; I − intensity with sound. Data are represented as mean ± SD.

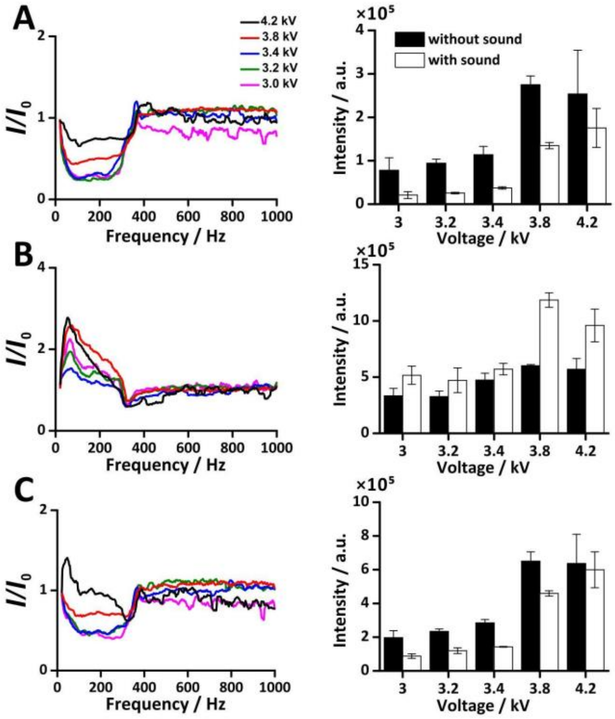

Figure S9. Influence of the voltage of ESI capillary on *EF* and intensity in the presence of sound. (A) *EF* (left) and intensity (right) for alanine (*m/z* 90; positive-ion mode) as a function of sinusoidal voltage frequency at different distances. (B) *EF* (left) and intensity (right) for lysine (*m/z* 147; positive-ion mode) as a function of sinusoidal voltage frequency at different distances. (C) *EF* (left) and intensity (right) for phenylalanine (*m/z* 166; positive-ion mode) as a function of sinusoidal voltage frequency at different distances. Sinusoidal voltage amplitude: 1 V. Sample concentration: 5 μM in 25% (v/v) methanol in water. Sample was injected into the ESI source by a peristaltic pump at a flow rate of 50 μL min^{-1}. The three replicates were averaged to generate *EF* plots. Intensities obtained with sound from 50 to 300 Hz were averaged, and shown in the bar plots. The number of averaged values was 248. Symbols: I_0 − intensity without sound; I − intensity with sound. Data are represented as mean ± SD.

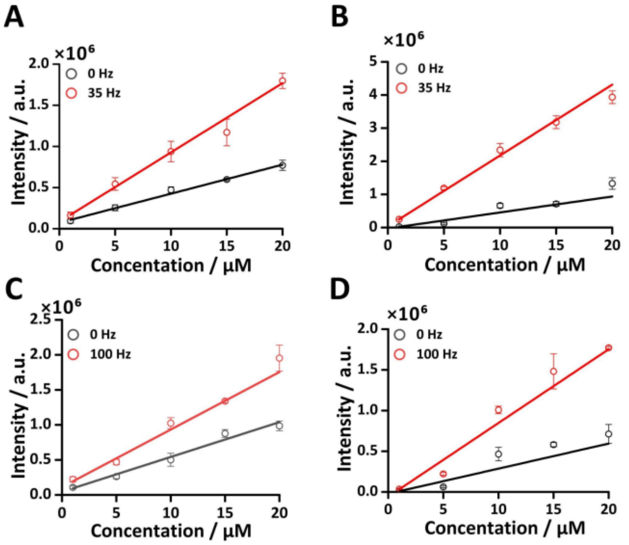

Figure S10. Calibration plots for the large woofer (A,B) and four small woofers (C,D). Sinusoidal voltage amplitude: 1V for the larger woofer, 3V for the four small woofers. Analytes: (A,C) GGY (*m/z* 296; positive-ion mode); (B,D) HPF7 (*m/z* 673; positive-ion mode; charge state: 4). The QqQ-MS was operated in SIM mode. Sample concentration: 1, 5, 10, 15, 20 μM in 25% (v/v) methanol in water containing 0.1% (v/v) formic acid. Data are represented as mean ± SD.

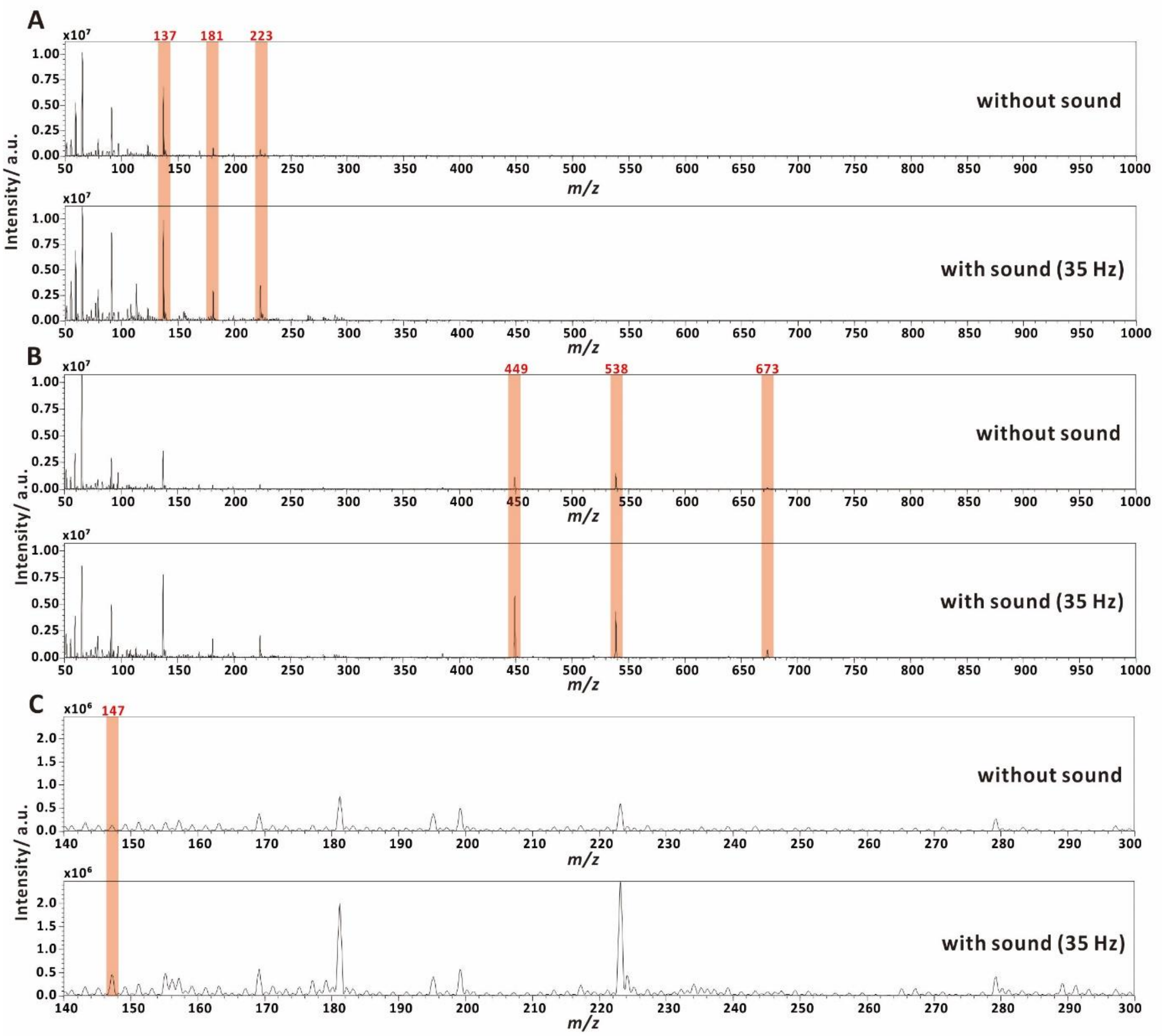

Figure S11. Mass spectra obtained during analysis of a real sample without and with sound (one large woofer). Liquid from facial mask was diluted 1000× with 25% (v/v) aqueous methanol and 0.1% formic acid. (A) Unspiked diluted extract (1 V, 35 Hz); (B) diluted extract spiked with 5 μM HPF7 (1 V, 35 Hz); (C) diluted extract spiked with and 5 μM lysine (1 V, 35 Hz).

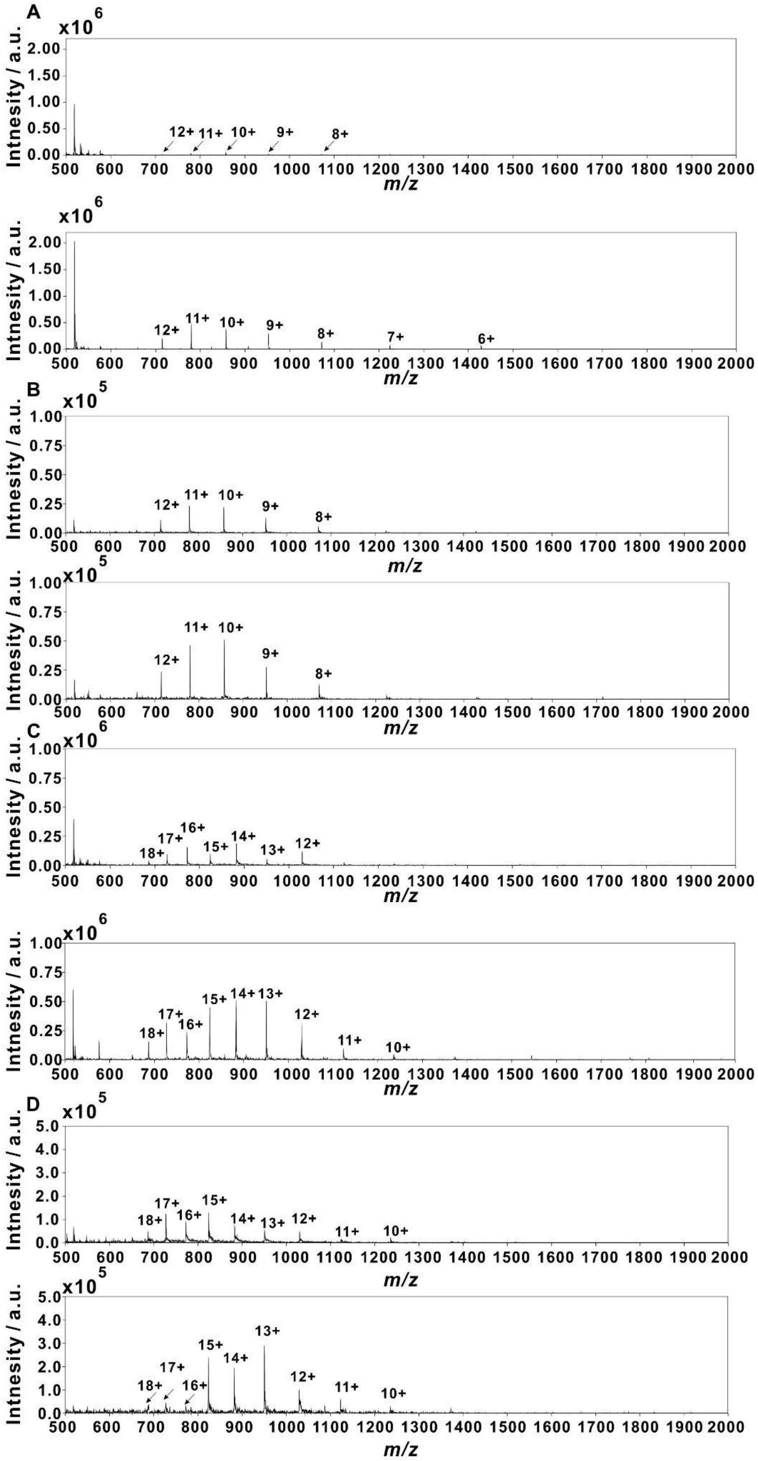

Figure S12. Mass spectra of proteins with (A,C) the larger woofer and (B,D) four small woofers. Analytes: (A,B) ubiquitin; (C,D) cytochrome *c*. Spectra obtained without application of sound (top), and spectra obtained with application of sound (200 Hz, 1 V for the larger woofer; 100 Hz, 3 V for the four small woofers; bottom). The QqQ-MS was operated in Q3 mode. Sample concentration: 5 μM in 25% (v/v) methanol in water containing 10 mM ammonium acetate and 0.1% (v/v) formic acid.

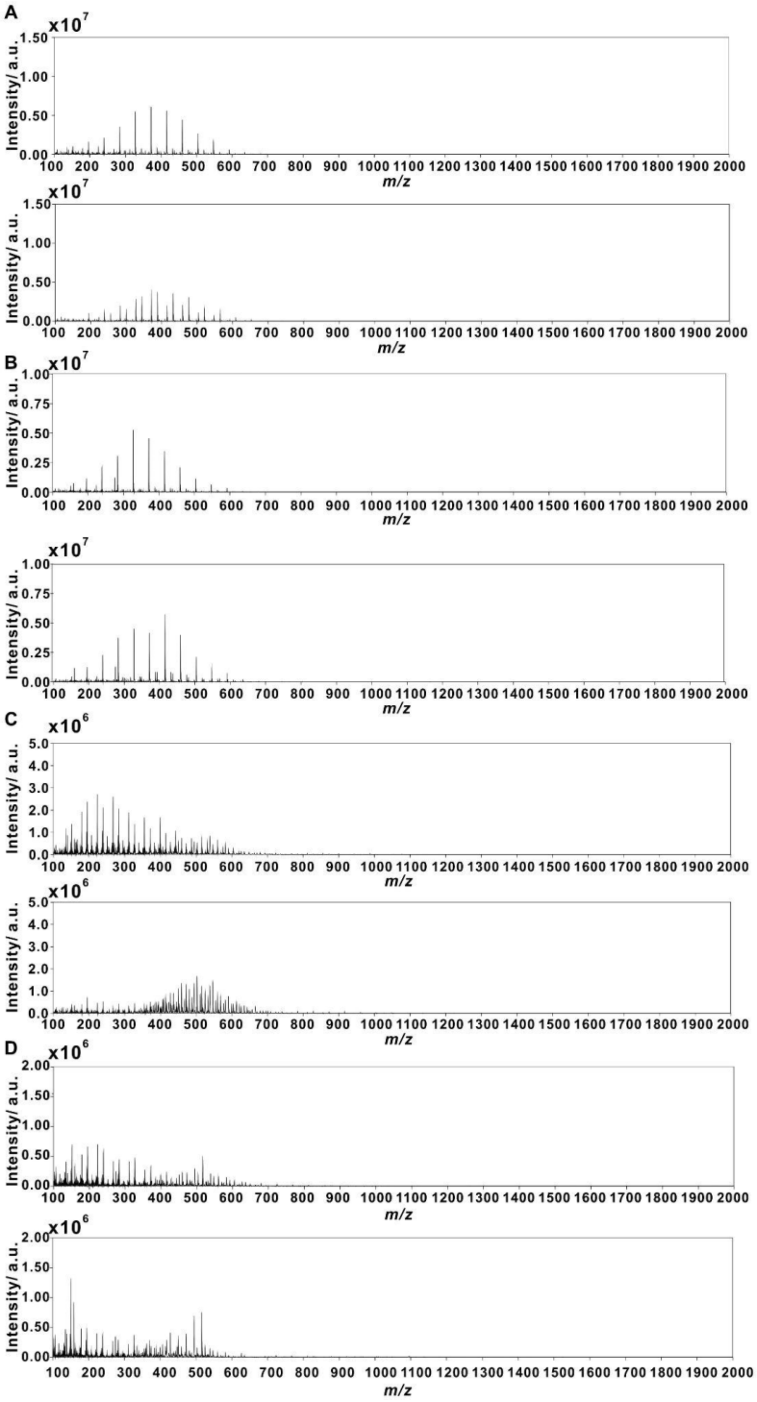

Figure S13. Mass spectra of PEG with (A,C) the larger woofer and (B,D) four small woofers. Analytes: (A,B) PEG 400; (C,D) PEG 1000. Spectra obtained without application of sound (top), and spectra obtained with application of sound (200 Hz, 1 V for the larger woofer; 100 Hz, 3 V for the four small woofers; bottom). The QqQ-MS was operated in Q3 mode. Sample concentration: 5 µM in 25% (v/v) methanol in water containing and 0.1% (v/v) formic acid.

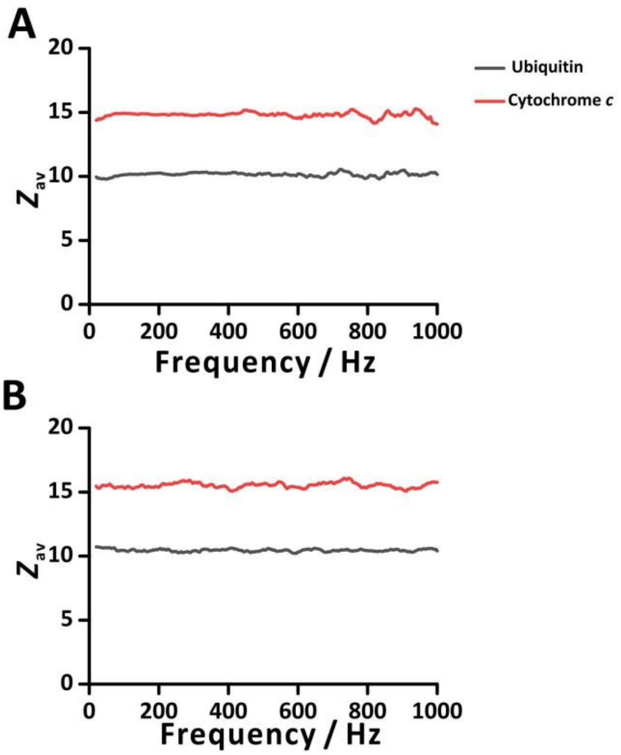

Figure S14. Weighted average charge state (Z_{av}) of proteins as a function of sinusoidal voltage frequency of the woofer: (A) One large woofer; (B) four small woofers with different proteins. Analytes: ubiquitin, cytochrome *c*. Sinusoidal voltage amplitude: 1 V for the large woofer, 3V for the four small woofers. The QqQ-MS was operated in Q3 mode. Sample concentration: 5 μM in 25% (v/v) methanol in water containing 10 mM ammonium acetate and 0.1% (v/v) formic acid. The Z_{av} of proteins were calculated using the formula: $Z_{av} = \Sigma(Z_i I)/\Sigma I$.

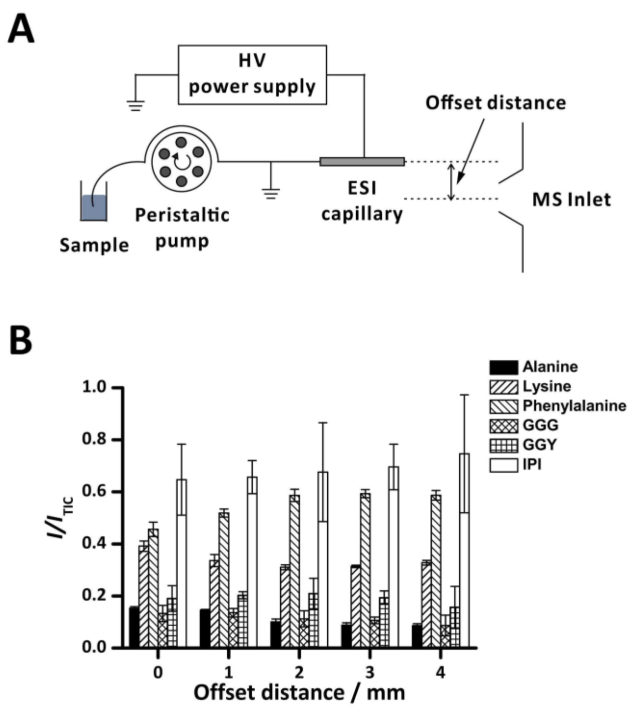

Figure S15. Influence of the offset distance between ESI capillary and MS inlet on MS signal intensity: (A) schematic diagram of experimental setups; (B) normalized intensities with different offset distances. Analytes: alanine (*m/z* 90); lysine (*m/z* 147); phenylalanine (*m/z* 166) in mixture; GGG (*m/z* 190); GGY (*m/z* 296); IPI (*m/z* 342) in mixture. Positive-ion SIM mode was used. Analyte concentration: 5 µM in 25% (v/v) methanol in water. Sample was injected into the ESI source by a peristaltic pump at a flow rate of 50 µL min^{-1}. The distance between ESI capillary and MS inlet was 8 mm. Symbols: I_{TIC} − intensity from TIC; I − intensity from EIC. The ion current was averaged from 1 min to 1.5 min. Data are represented as mean ± SD.

Methods S1. Computer codes for data acquisition and processing

Image processing (Python)

```
# Import libraries
import os
import cv2
import tkinter as tk
from tkinter import filedialog as fd
import time

# Graphical user interface
top = tk.Tk()
top.title('ESI image processing')

frame = tk.Frame(top)
frame.pack()
space = tk.Label(frame, text = "        ", width = 35, height = 3)
space.pack()

def getFolder():
    top.dir = fd.askdirectory()
space = tk.Button(frame, text = "Select Folder", command = getFolder)
space.pack(side = tk.TOP)

frame = tk.Frame(top)
frame.pack()
space = tk.Label(frame, text = "        ", width = 35, height = 3)
space.pack()

frame = tk.Frame(top)
frame.pack()
space = tk.Button(frame, text = "OK", command = top.destroy)
space.pack(side = tk.TOP)

frame = tk.Frame(top)
frame.pack()
space = tk.Label(frame, text = "        ", width = 35, height = 3)
space.pack()

top.mainloop()

# Process images
count = 1
Folder = top.dir
for i, filename in enumerate(os.listdir(Folder)):
    # Rename file
    Folder = top.dir
    new_name = 'a'
    renamed_file = new_name + str(count).zfill(5) + ".bmp"
```

```
    source_file = Folder + "/" + filename
    renamed_file = Folder + "/" + renamed_file
    os.rename(source_file, renamed_file)

    # Import image
    img = cv2.imread(Folder + '/a' + str(count).zfill(5) + '.bmp',0)

    # Adaptive thresholding
    imgBlur = cv2.medianBlur(img,9)
    threshold_img =
cv2.adaptiveThreshold(imgBlur,255,cv2.ADAPTIVE_THRESH_GAUSSIAN_C,cv2.THRESH
_BINARY,51,3)

    # Save the processed image
    cv2.imwrite(Folder + '/a' + str(count).zfill(5) + '_processed.jpg',
threshold_img)

    # Convert original image from bmp to jpg format
    cv2.imwrite(Folder + '/a' + str(count).zfill(5) + '.jpg', img)
    os.remove(Folder + '/a' + str(count).zfill(5) + '.bmp')
    count += 1

# Task completion message
message = tk.Tk()
message.title('ESI image processing')
frame4 = tk.Frame(message)
frame4.pack()
Text = tk.Label(frame4, text = "Image processing successful.", width = 50,
height = 10)
Text.pack()
message.mainloop()

# References:
'''1. OpenCV - Adaptive Thresholding,
https://docs.opencv.org/master/d7/d4d/tutorial_py_thresholding.html
   2. OpenCV - Miscellaneous Image Transformations,
https://docs.opencv.org/master/d7/d1b/group__imgproc__misc.html
   3. OpenCV - Flags used for image file reading and writing,
https://docs.opencv.org/3.4/d8/d6a/group__imgcodecs__flags.html
   4. Wang, Y.-W.; Prabhu, G. R. D.; Hsu, C.-Y.; Urban, P. L. Tuning
Electrospray Ionization with Low-Frequency Sound. J. Am. Soc. Mass
Spectrom. 2022, 33, 1883-1890.'''
```

Macro for analyzing particles (ImageJ Macro Language)

```
run("Set Scale...", "distance=339.4186 known=1000 unit=um");
setAutoThreshold("Default dark no-reset");
//run("Threshold...");
setThreshold(0, 30, "raw");
run("Close");
setOption("BlackBackground", true);
run("Convert to Mask", "background=Dark calculate black");
close;
run("Set Measurements...", "area mean min center shape redirect=None
decimal=3");
run("Analyze Particles...", "size=100-12500 circularity=0.30-1.00 display
exclude clear summarize add stack");
```

Frequency sweeping for the setup with one large woofer (JavaScript)

```
clear()
if(!('StaticIO' in this)) throw "Please open the StaticIO instrument";
if(!('Wavegen' in this) || !('Scope' in this)) throw "Please open a Scope
and a Wavegen instrument";
print("Running StaticIO script");
StaticIO.Channel0.Mode.text = "IOs";
StaticIO.Channel0.DIO7.Mode.text = "Button";
StaticIO.Channel0.DIO7.text = "1";
wait(1); // Unit: s
StaticIO.Channel0.DIO7.text = "0";
wait(90); // Unit: s
Wavegen.Channel1.Mode.text = "Sweep";
Wavegen.Channel1.Sweep.Type.text = "Sine";
Wavegen.Channel1.Sweep.Frequency.Start.value = 20; // Unit: Hz
Wavegen.Channel1.Sweep.Frequency.Stop.value = 1000; // Unit: Hz
Wavegen.Channel1.Sweep.Frequency.Time.value = 300; // Unit: s
Wavegen.Channel1.Sweep.Amplitude.Start.value = 1; // Unit: V
Wavegen.Channel1.Sweep.Offset.value = 0; // Unit: V
Wavegen.Channel1.Sweep.Symmetry.value = 50; // Unit: %
Wavegen.Channel1.Sweep.Phase.value = 0; // Unit: ?
Scope.Trigger.Trigger.text = "Repeated";
Wavegen.run();
Scope.run();
for(var i = 0; i < 50; i++){
print(i+1, "s");
wait(1);
}

# References:
'''1. Wang, Y.-W.; Prabhu, G. R. D.; Hsu, C.-Y.; Urban, P. L. Tuning
Electrospray Ionization with Low-Frequency Sound. J. Am. Soc. Mass
Spectrom. 2022, 33, 1883-1890.'''
```

Frequency sweeping for the setup with four small woofers (Python)

```
import tkinter as tk
from tkinter import messagebox
from ctypes import *
import sys
import time
from os import sep

if sys.platform.startswith("win"):
    dwf = cdll.LoadLibrary("dwf.dll")
    constants_path = "C:" + sep + "Program Files (x86)" + sep + "Digilent"
+ sep + "WaveFormsSDK" + sep + "samples" + sep + "py"
elif sys.platform.startswith("darwin"):
    dwf = cdll.LoadLibrary("/Library/Frameworks/dwf.framework/dwf")
else:
    dwf = cdll.LoadLibrary("libdwf.so")

# import constants
sys.path.append(constants_path)
from dwfconstants import *

# check library loading errors
szerr = create_string_buffer(512)
dwf.FDwfGetLastErrorMsg(szerr)
print(szerr.value)

# declare ctype variables
IsInUse = c_bool()
hdwf = c_int()
rghdwf = []
cChannel = c_int()
cDevices = c_int()
voltage = c_double()
sts = c_byte()
dwRead = c_uint32()

# declare string variables
devicename = create_string_buffer(64)
serialnum = create_string_buffer(16)

# print DWF version
version = create_string_buffer(16)
dwf.FDwfGetVersion(version)
print("DWF Version: " + version.value.decode('utf-8'))

# enumerate connected devices
dwf.FDwfEnum(c_int(0), byref(cDevices))
if cDevices.value == 0:
    print("No device found")
    dwf.FDwfDeviceCloseAll()
    sys.exit(0)
```

```
print("Number of Devices: " + str(cDevices.value))

# open devices
for iDevice in range(0, cDevices.value):
    dwf.FDwfEnumDeviceName(c_int(iDevice), devicename)
    dwf.FDwfEnumSN(c_int(iDevice), serialnum)
    print("------------------------------")
    print("Device " + str(iDevice + 1) + " : \t" +
devicename.value.decode('utf-8') + "\t" + serialnum.value.decode(
        'utf-8'))
    dwf.FDwfDeviceOpen(c_int(iDevice), byref(hdwf))
    rghdwf.append(hdwf.value)

    if hdwf.value == 0:
        szerr = create_string_buffer(512)
        dwf.FDwfGetLastErrorMsg(szerr)
        print(szerr.value)
        dwf.FDwfDeviceCloseAll()
        sys.exit(0)

print(rghdwf)

# Graphical User Interface
win = tk.Tk()
win.title("Small woofer setting")
win.geometry("400x400+550+150")
win.config(background= "#323232")

#Sound parameters setting
hzStart  = ''
hzStop  = ''
secSweep = ''
amp = ''
hzStart_insert = '20' # unit: Hz
hzStop_insert = '1000' # unit: Hz
secSweep_insert = '300' # unit: s
amp_insert = '3' # unit: V

#function
def yes_or_yes():
    global hzStart, hzStop, secSweep, amp, hzMid
    try:
        hzStart = float(en_hzStart.get())
        hzStop = float(en_hzStop.get())
        secSweep = float(en_secSweep.get())
        amp = float(en_amp.get())
        hzMid = float(hzStart+hzStop)/2
        win.destroy()
        print("Sweeping frequency: ",float(hzStart), "Hz", "to",
float(hzStop), "Hz", "\n", "sweeping time: ", float(secSweep), "s","\n",
float(amp),"V", "\n" ,"hzMid: ", float(hzMid), "Hz")
        print("------------------------------")
```

```
    except ValueError:
        messagebox.showerror("Error", "ValueError！")


#GUI
lb_hzStart = tk.Label(bg = "#323232", fg = "white", text = 'hzStart / Hz')
lb_hzStart.config(font = "Calibri 12 bold")
lb_hzStart.pack()

en_hzStart = tk.Entry()
en_hzStart.pack()
en_hzStart.insert(0,hzStart_insert)



lb_hzStop = tk.Label(bg = "#323232", fg = "white", text = ' hzStop / Hz')
lb_hzStop.config(font = "Calibri 12 bold")
lb_hzStop.pack()

en_hzStop = tk.Entry()
en_hzStop.pack()
en_hzStop.insert(0,hzStop_insert)


lb_secSweep = tk.Label(bg = "#323232", fg = "white", text = ' secSweep /
s')
lb_secSweep.config(font = "Calibri 12 bold")
lb_secSweep.pack()

en_secSweep = tk.Entry()
en_secSweep.pack()
en_secSweep.insert(0,secSweep_insert)

lb_blank = tk.Label(bg = "#323232",text = '     ')
lb_blank.pack()

lb_amp = tk.Label(bg = "#323232", fg = "white", text = 'Woffer amplitude/
V')
lb_amp.config(font = "Calibri 12 bold")
lb_amp.pack()

en_amp = tk.Entry()
en_amp.pack()
en_amp.insert(0,amp_insert)



btn = tk.Button(text = "Yes or Yes",bg = "white",command = yes_or_yes)
btn.config(font = "Calibri 12 bold")
btn.pack(pady=10)

win.mainloop()
```

```
# Static IO
for iDevice in range(len(rghdwf)):
     hdwf.value = rghdwf[iDevice]
     if iDevice == 0:
         # enable output/mask on 8 LSB IO pins, from DIO 0 to 7
         dwf.FDwfDigitalIOOutputEnableSet(hdwf, c_int(0x00FF))
         dwf.FDwfDigitalIOOutputSet(hdwf, c_int(0x0080))
         dwf.FDwfDigitalIOStatus(hdwf)
         dwf.FDwfDigitalIOInputStatus(hdwf, byref(dwRead))
         print("------------------------------")
         print("Static IO starts!")
         print("Device: "+str(iDevice+1)+"\t"+"DIO: 7")
         print("Digital IO Pins: ", bin(dwRead.value)[2:].zfill(16))
         time.sleep(0.1) # unit: s
         dwf.FDwfDigitalIOOutputEnableSet(hdwf, c_int(0x0080))
         dwf.FDwfDigitalIOOutputSet(hdwf, c_int(0x0000))
         dwf.FDwfDigitalIOInputStatus(hdwf, byref(dwRead))
         print("Digital IO Pins: ", bin(dwRead.value)[2:].zfill(16))
         print("------------------------------")
         time.sleep(90)

#Wavegen
for iDevice in range(len(rghdwf)):
    hdwf.value = rghdwf[iDevice]
    if iDevice == 0:
        dwf.FDwfDeviceAutoConfigureSet(hdwf, c_int(0))
dwf.FDwfAnalogOutNodeEnableSet(hdwf, c_int(-1), AnalogOutNodeCarrier,
c_bool(True))
        dwf.FDwfAnalogOutNodeFunctionSet(hdwf, c_int(-1),
AnalogOutNodeCarrier, funcSine)
        dwf.FDwfAnalogOutNodeOffsetSet(hdwf, c_int(-1),
AnalogOutNodeCarrier, c_double(0))

        dwf.FDwfAnalogOutNodeEnableSet(hdwf, c_int(-1), AnalogOutNodeFM,
c_int(1))
        dwf.FDwfAnalogOutNodeFunctionSet(hdwf, c_int(-1), AnalogOutNodeFM,
funcRampUp)
        dwf.FDwfAnalogOutNodeFrequencySet(hdwf, c_int(-1), AnalogOutNodeFM,
c_double(1.0/secSweep))
        dwf.FDwfAnalogOutNodeAmplitudeSet(hdwf, c_int(-1), AnalogOutNodeFM,
c_double(100.0*(hzStop-hzMid)/hzMid))
        dwf.FDwfAnalogOutNodeSymmetrySet(hdwf, c_int(-1), AnalogOutNodeFM,
c_double(100))
        #Woffer 1,2
        dwf.FDwfAnalogOutNodeFrequencySet(hdwf, c_int(-1),
AnalogOutNodeCarrier, c_double(hzMid))
        dwf.FDwfAnalogOutNodeAmplitudeSet(hdwf, c_int(-1),
AnalogOutNodeCarrier, c_double(amp))

        dwf.FDwfAnalogOutNodePhaseSet(hdwf, c_int(0), AnalogOutNodeCarrier,
c_double(0)) # CH1
```

```
        dwf.FDwfAnalogOutNodePhaseSet(hdwf, c_int(1), AnalogOutNodeCarrier, 
c_double(0)) # CH2
    if iDevice == 1:
        dwf.FDwfDeviceAutoConfigureSet(hdwf, c_int(0))
        dwf.FDwfAnalogOutNodeEnableSet(hdwf, c_int(-1), 
AnalogOutNodeCarrier, c_bool(True))
        dwf.FDwfAnalogOutNodeFunctionSet(hdwf, c_int(-1), 
AnalogOutNodeCarrier, funcSine)
        dwf.FDwfAnalogOutNodeOffsetSet(hdwf, c_int(-1), 
AnalogOutNodeCarrier, c_double(0))

        dwf.FDwfAnalogOutNodeEnableSet(hdwf, c_int(-1), AnalogOutNodeFM, 
c_int(1))
        dwf.FDwfAnalogOutNodeFunctionSet(hdwf, c_int(-1), AnalogOutNodeFM, 
funcRampUp)
        dwf.FDwfAnalogOutNodeFrequencySet(hdwf, c_int(-1), AnalogOutNodeFM, 
c_double(1.0/secSweep))
        dwf.FDwfAnalogOutNodeAmplitudeSet(hdwf, c_int(-1), AnalogOutNodeFM, 
c_double(100.0*(hzStop-hzMid)/hzMid))
        dwf.FDwfAnalogOutNodeSymmetrySet(hdwf, c_int(-1), AnalogOutNodeFM, 
c_double(100))
        #Woffer 3,4
        dwf.FDwfAnalogOutNodeFrequencySet(hdwf, c_int(-1), 
AnalogOutNodeCarrier, c_double(hzMid))
        dwf.FDwfAnalogOutNodeAmplitudeSet(hdwf, c_int(0), 
AnalogOutNodeCarrier, c_double(amp))
        dwf.FDwfAnalogOutNodeAmplitudeSet(hdwf, c_int(1), 
AnalogOutNodeCarrier, c_double(3))

        dwf.FDwfAnalogOutNodePhaseSet(hdwf, c_int(0), AnalogOutNodeCarrier, 
c_double(0)) # CH3
        dwf.FDwfAnalogOutNodePhaseSet(hdwf, c_int(1), AnalogOutNodeCarrier, 
c_double(0)) # CH4

        print("Generating sine wave...")

dwf.FDwfAnalogOutRunSet(1, c_int(-1), c_double(secSweep))
dwf.FDwfAnalogOutRunSet(2, c_int(-1), c_double(secSweep))
dwf.FDwfAnalogOutConfigure(1, c_int(-1), c_int(1))
dwf.FDwfAnalogOutConfigure(2, c_int(-1), c_int(1))
time.sleep(secSweep)

# Colse devices
dwf.FDwfDeviceCloseAll()
sys.exit(0)


# References:
'''1. WaveForms SDK Reference Manual - 3.22.2, 
https://files.digilent.com/manuals/WaveFormsSDK/3.22.2/WaveForms%20SDK%20Re
ference%20Manual.pdf
   2. The GitHub material, 
Windows 32-bit: C:\Program Files\Digilent\WaveFormsSDK\samples
```

```
Windows 64-bit: C:\Program Files (x86)\Digilent\WaveFormsSDK\samples
Linux: /usr/share/digilent/waveforms/samples
macOS: /Applications/WaveForms.app/Contents/Resources/SDK/samples '''
```
